# Influence of weakly coordinating anions binding to the hexa-*tert*-butyl dysprosocenium cation†

**DOI:** 10.1039/d4dt02713b

**Published:** 2024-11-05

**Authors:** Sophie C. Corner, Gemma K. Gransbury, David P. Mills

**Affiliations:** a Department of Chemistry, The University of Manchester Oxford Road Manchester M13 9PL UK david.mills@manchester.ac.uk gemmagransbury@gmail.com

## Abstract

Complexes containing isolated dysprosocenium cations, [Dy(Cp^R^)_2_][WCA] (Cp^R^ = substituted cyclopentadienyl, WCA = weakly coordinating anion), have recently emerged as leading examples of high-temperature single-molecule magnets (SMMs) due to a combination of the axial orientation and rigidity of the Cp^R^ rings. However, our understanding of the effects of transverse fields on the magnetic properties of [Dy(Cp^R^)_2_]^+^ cations is underdeveloped. Here we investigate the impact of equatorially-bound WCAs *via* the synthesis of the Dy(iii) bis-Cp^R^ complexes [Dy(Cp^ttt^)_2_{AlCl[OC(CF_3_)_3_]_3_-κ-*Cl*}] (1) and [Dy(Cp^ttt^)_2_{AlCl(C_2_H_5_)[OC(C_6_F_5_)_3_]_2_-κ-*Cl*}] (2), and their characterisation by single crystal XRD, elemental analysis, ATR-IR and NMR spectroscopy, and *ab initio* calculations. Despite the similarity of the Dy coordination spheres in 1 and 2 we find that their effective energy barriers to reversal of magnetisation are vastly different (*U*_eff_ = 886(17) cm^−1^ and 559(18) cm^−1^, respectively) and they both show waist-restricted magnetic hysteresis at 2 K. Together, these data provide fresh insights into the sensitivity of the magnetic properties of [Dy(Cp^R^)_2_]^+^ cations to relatively weak equatorial interactions.

## Abstract 2

We present the synthesis and characterisation of two dysprosium metallocene complexes with bound weakly coordinating anions. Despite their similarity these complexes exhibit vastly different effective energy barriers to magnetic reversal.

## Graphical abstract

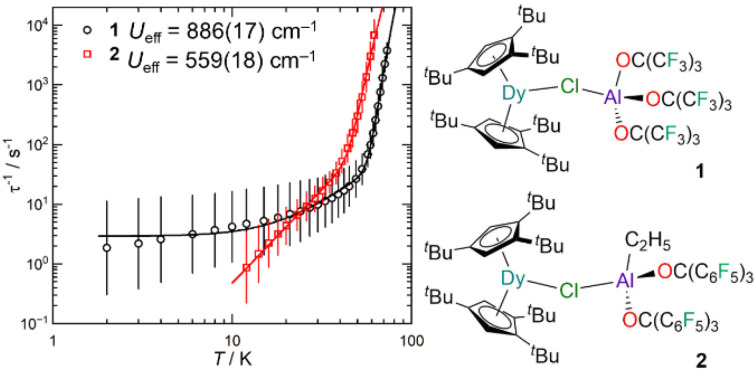

## Introduction

Single-molecule magnets (SMMs) show effective energy barriers to the relaxation of magnetisation (*U*_eff_) and can exhibit open magnetic hysteresis up to a certain temperature (*T*_H_);^1^ lanthanide (Ln) complexes have provided the most promising SMM candidates to date.^2,3^ Dy(iii) and Tb(iii) SMMs with axial crystal fields have been targeted most often as these geometries provide the largest *U*_eff_ through stabilisation of the ground ±*m*_*J*_ states and concomitant destabilisation of the least magnetic ±*m*_*J*_ states for these ions.^4–6^ Ideal Dy(iii) and Tb(iii) complexes with perfectly linear geometries would exhibit suppressed Raman and quantum tunnelling of magnetisation (QTM) under-barrier relaxation mechanisms due to high ±*m*_*J*_ state purities,^4–6^ but such complexes are difficult to achieve as predominantly ionic Ln bonding regimes favour additional metal–ligand interactions.^7^

In 2017 the first isolated dysprosocenium complex, [Dy(Cp^ttt^)_2_][B(C_6_F_5_)_4_] (Cp^ttt^ = C_5_H_2_^*t*^Bu_3_-1,2,4), was reported to exhibit a large *U*_eff_ value of 1223(14) cm^−1^ and *T*_H_ of 60 K due to a combination of its axial geometry and the rigid aromatic Cp^ttt^ rings suppressing Raman and QTM processes.^8^ It is noteworthy that the precursor to this complex, [Dy(Cp^ttt^)_2_(Cl)] is not an SMM in zero field and has *T*_H_ < 2 K,^9–11^ highlighting the substantial impact of equatorially-bound ligands on the magnetic properties of complexes containing {Dy(Cp^R^)_2_} (Cp^R^ = substituted cyclopentadienyl) cores, as previously shown systematically for halides in [Dy(Cp*)_2_(X)(THF)] (Cp* = C_5_Me_5_, X = Cl, Br, I).^12^ In the interim many other high-temperature SMMs have been synthesised that contain {Dy(Cp^R^)_2_} (Cp^R^ = substituted cyclopentadienyl) motifs,^13–15^ or heteroatom-containing analogues,^16–19^ and the expansion of this chemistry to dinuclear complexes has since provided the current best-performing Dy Cp^R^-based SMM to date, [{Dy(C_5_^i^Pr_5_)}_2_(μ-I)_3_], (*U*_eff_ = 1631(25) cm^−1^; *T*_H_ = 80 K).^20^

Recently, we reported that a Dy(iii) contact ion-pair complex containing a weakly coordinating anion (WCA), [Dy(Cp^ttt^)(Cp*){Al[OC(CF_3_)_3_]_4_-κ-*F*}], maintains a high *U*_eff_ = 1265(15) cm^−1^, but its *T*_H_ value (36 K) is significantly diminished compared to the corresponding separated ion-pair complex [Dy(Cp^ttt^)(Cp*)] [Al{OC(CF_3_)_3_}_4_] (*U*_eff_ = 1221(25) cm^−1^, *T*_H_ = 52 K), despite the geometrical similarity of their {Dy(Cp^ttt^)(Cp*)} cores.^15^ Conversely, the related halobenzene-bound complexes [Dy(Cp^ttt^)(Cp*)(XPh-κ-*X*)][Al{OC(CF_3_)_3_}_4_] (X = F, Cl, Br) exhibit lower *U*_eff_ (range: 1100(9)–1182(9) cm^−1^) and *T*_H_ (range: 22–24 K) values,^21^ challenging assumptions that an anion always introduces a greater transverse field than a weakly-bound neutral ligand.^12,22–42^ We sought to investigate the effect of other WCAs binding to {Dy(Cp^ttt^)_2_} to further establish the sensitivity of SMM parameters to weak equatorial interactions.

Here we disclose the synthesis of [Dy(Cp^ttt^)_2_{AlCl[OC(CF_3_)_3_]_3_-κ-*Cl*}] (1) and [Dy(Cp^ttt^)_2_{AlCl(C_2_H_5_)[OC(C_6_F_5_)_3_]_2_-κ-*Cl*}] (2), and their characterisation by ATR-IR and NMR spectroscopy, elemental analysis, SQUID magnetometry and complete active space self-consistent field spin–orbit (CASSCF-SO) calculations. We find that coordination of the WCA to the Dy(iii) ion in these complexes increases Dy⋯Cp^ttt^ distances and reduces Cp^ttt^_centroid_⋯Dy⋯Cp^ttt^_centroid_ angles *vs*. parent [Dy(Cp^ttt^)_2_][B(C_6_F_5_)_4_]^8^ to significantly reduce the strength of the axial ligand field. The purities of the ±*m*_*J*_ state manifold are reduced to different extents in 1 and 2 but in both cases *U*_eff_ and *T*_H_ are hugely diminished *cf*. [Dy(Cp^ttt^)_2_][B(C_6_F_5_)_4_],^8^ with the differences in *U*_eff_ highlighting the extreme sensitivity of the electronic structures of Dy(iii) ions to the variable strength of WCA binding.

## Results and discussion

### Synthesis

**Fig. 1:**
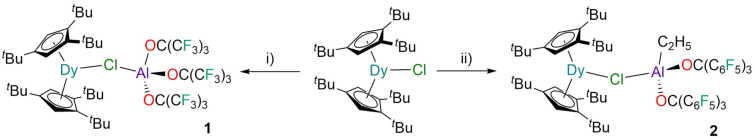
Synthesis of 1 and 2. Conditions: (i) PhF-Al{OC(CF_3_)_3_}_3_ in fluorobenzene at room temperature; (ii) [Al(C_2_H_5_){OC(C_6_F_5_)_3_}_2_] in fluorobenzene at room temperature.

The reaction of [Dy(Cp^ttt^)_2_(Cl)]^8,10,11^ with one equivalent of PhF-Al{OC(CF_3_)_3_}_3 _^43^ in fluorobenzene gave bright yellow crystals of 1 in 58% yield after recrystallisation from pentane (Fig. 1). The addition of a further equivalent of PhF-Al{OC(CF_3_)_3_}_3_ did not lead to the expected abstraction of chloride to form [Dy(Cp^ttt^)_2_][{Al[OC(CF_3_)_3_]_3_}_2_(μ-Cl)],^44^*via in situ*-generation of a bulkier WCA. Similarly, the reaction of [Dy(Cp^ttt^)_2_(Cl)] with [Al(C_2_H_5_){OC(C_6_F_5_)_3_}_2_] in fluorobenzene gave yellow crystals of 2 in 60% yield after recrystallisation (Fig. 1). This superacid was chosen as [Dy(Cp^ttt^)_2_(Cl)] did not react with [Al{OC(C_6_F_5_)_3_}_3_]^45^ in fluorobenzene at room temperature. The stoichiometric reaction of 2 with HOC(C_6_F_5_)_3_ in hexane gave [Dy(Cp^ttt^)_2_(Cl)] and [Al{OC(C_6_F_5_)_3_}_3_], as a stronger thermodynamic driving force is required for chloride abstraction from the highly Lewis acidic Dy(iii) centre.^8,10,11^

### Bulk characterisation

Elemental analysis results obtained for 1 and 2 typically gave lower carbon and hydrogen values than expected, which we attribute to carbide formation^46^ and the uncertainty that can be introduced in these experiments from high fluorine contents.^47^ The ATR-IR spectra of 1 and 2 show diagnostic resonances for C–H, C–F and C–O stretches (see ESI Fig. S1 and S2†). The paramagnetism of 1 and 2 preclude the assignment of their ^1^H, ^13^C{^1^H} and ^19^F NMR spectra (Fig. S3 and S5†); the magnetic susceptibility values obtained by the Evans method^48^ in fluorobenzene solutions (with a C_6_H_5_F/C_6_D_6_ insert; Fig. S4 and S6†) are close to the expected value for a Dy(iii) ion (1: 10.63*μ*_B_, 14.1 cm^3^ K mol^−1^, 2: 10.20*μ*_B_, 13.0 cm^3^ K mol^−1^; expected: 10.65*μ*_B_, 14.2 cm^3^ K mol^−1^).^49^

### Single crystal X-ray crystallography

**Fig. 2:**
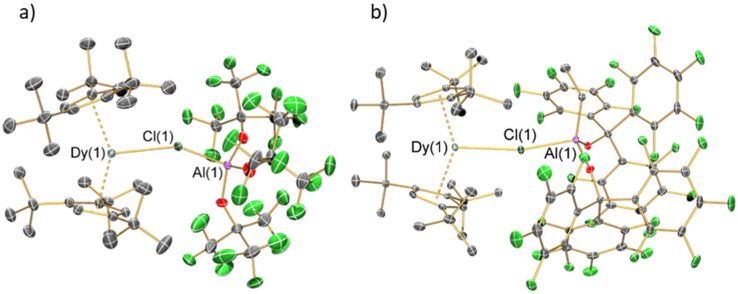
Single crystal XRD structures of (a) 1 and (b) 2 (Dy: cyan, C: grey, Al: purple, F: green, O: red). Displacement ellipsoids set at 30% probability levels; hydrogen atoms and the lattice fluorobenzene molecules have been omitted for clarity.

The solid-state structures of 1 and 2 were determined by single crystal XRD (structures depicted in Fig. 2, selected bond distances and angles compiled in Table 1; for crystallographic parameters see ESI Table S1†). These complexes show similar bulk features, with *pseudo*-trigonal arrangements of the two Cp^ttt^ centroids and Cl. Both complexes exhibit significant disorder at 100 K; for 1 this is observed in the {Cl-Al[OC(CF_3_)_3_]_3_} moiety and parameters considered are the mean values for the disordered components, whilst for 2 there is disorder of the Cp^ttt^ groups about the Dy(iii) centre in the plane perpendicular to the Dy–Cl axis and only the component with the highest occupancy is considered. The Cp^ttt^_centroid_⋯Dy⋯Cp^ttt^_centroid_ angles of 1 [145.71(2)°] and 2 [145.57(2)°] are more bent than in both [Dy(Cp^ttt^)_2_][B(C_6_F_5_)_4_] [152.56(7)°] and [Dy(Cp^ttt^)_2_(Cl)] [146.67(7)°];^8,10,11^ this is in accord with the large steric effects imposed by the WCAs. The proximity of the WCAs in 1 and 2 also induces an eclipsed arrangement of the Cp^ttt^ groups (Fig. S10 and S11†); by contrast [Dy(Cp^ttt^)_2_(Cl)] and [Dy(Cp^ttt^)_2_][B(C_6_F_5_)_4_] both adopt a staggered arrangement.^8,10,11^ The Dy–Cl–Al angle of 1 [152.1(5)°] is lower than that of 2 [165.30(7)°], due to the three {OC(CF_3_)_3_} substituents in the former compared to one ethyl and two {OC(C_6_F_5_)_3_} groups in the latter showing different steric effects.

**Table 1:** Selected bond lengths and angles of 1 and 2

Parameter	1	2
Dy(1)⋯Cp^ttt^_centroid_	2.3580(6) Å	2.3783(2) Å
Dy(1)⋯Cp^ttt^_centroid_	2.3517(8) Å	2.3532(3) Å
Dy–Cl(1)	2.801(9) Å	2.7301(12) Å
Closest Dy(1)⋯C	2.979(10) Å	3.002(6) Å
Closest Dy(1)⋯H	2.562 Å	2.765 Å
Al(1)–Cl(1)	2.220(10) Å	2.316(2) Å
Cp^ttt^_centroid_⋯Dy(1) ⋯Cp^ttt^_centroid_	145.71(2)°	145.57(2)°
Cp^ttt^_centroid_⋯Dy(1)–Cl(1)	100.61(9)°	107.87(3)°
Cp^ttt^_centroid_⋯Dy(1)–Cl(1)	112.49(9)°	106.46(3)°
Dy(1)–Cl(1)–Al(1)	152.1(5)°	165.30(7)°

The mean Dy⋯Cp^ttt^ distances of 1 [2.3549(10) Å] and 2 [2.3658(4) Å] are longer than in [Dy(Cp^ttt^)_2_][B(C_6_F_5_)_4_] [2.316(3) Å] but shorter than for [Dy(Cp^ttt^)_2_(Cl)] [2.413(3) Å].^8,10,11^ This can be attributed to the Dy–Cl bond distance in [Dy(Cp^ttt^)_2_(Cl)] [2.5480(12) Å] being shorter than for the WCAs in 1 [2.801(9) Å] and 2 [2.7301(12) Å]. Following correction for relative covalent radii (F: 0.64 Å; Cl: 0.99 Å),^50^ the Dy–Cl bond lengths in 1 [1.811(9) Å] and 2 [1.7401(12) Å] are shorter than the Dy–F bond in [Dy(Cp^ttt^)(Cp*){Al[OC(CF_3_)_3_]_4_-κ-*F*}] [2.812(4) Å before and 2.172(4) Å after correction];^15^ this indicates that the WCAs in 1 and 2 bind strongly. The longer Dy–Cl distance in 1 compared to 2 can be ascribed to a combination of steric and electronic effects, where the combination of small ethyl and bulky {OC(C_6_F_5_)_3_} groups of 2 lower the Lewis acidity and coordinative saturation of the Al centre; this is also reflected in the increased Al–Cl bond distance from 1 [2.220(10) Å] to 2 [2.316(2) Å].

### Magnetism and CASSCF-SO calculations

**Fig. 3:**
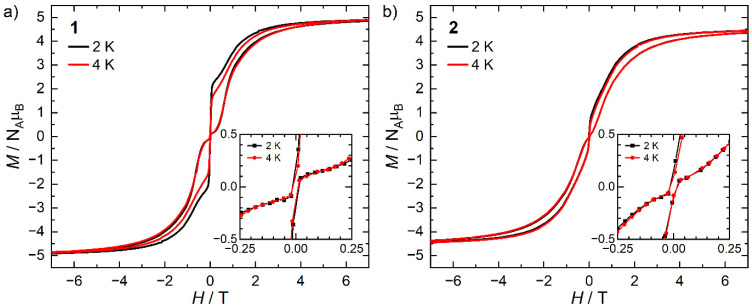
*M vs. H* hysteresis loops of (a) 1 and (b) 2 suspended in eicosane at 2 and 4 K, inset shows zero field region. The sweep rates are 22 Oe s^−1^ for |*H*| < 1 T, 54 Oe s^−1^ for 1 < |*H*| < 2 T, and 91 Oe s^−1^ for 2 < |*H*| < 7 T.

The static and dynamic magnetic properties of 1 and 2 were investigated by SQUID magnetometry (Fig. 3, ESI Fig. S12–S23 and Tables S2 and S3†). The molar magnetic susceptibilities (*χ*_M_*T*) at 300 K [1: 13.2 cm^3^ K mol^−1^; 2: 12.1 cm^3^ K mol^−1^] are lower than the expected Dy(iii) free-ion value (^6^H_15/2_, *χT* = 14.2 cm^3^ K mol^−1^)^51^ and the Evans method values, which we attribute to small mass or sample shape correction errors (measurement of sample dimensions and assumption of a cylindrical sample). The susceptibilities decrease slowly with temperature to *ca*. 22 K for 1 and 26 K for 2, at which point there is a sharp decrease that can be attributed to slow thermalisation of the sample. Saturation of the spin states *via* the application of a 7 T field at 2 K results in a magnetisation of 4.92 *Nμ*_B_ for 1 and 4.46 *Nμ*_B_ for 2; the latter is markedly lower than the expected value of *ca*. 5 *Nμ*_B_, which is also attributed to the mass or shape correction error.^52^ Magnetisation *vs.* field measurements are the first indication of slow magnetisation dynamics with a step at low fields.^53^ Butterfly-shaped hysteresis is observed for both complexes (Fig. 3), with 1 retaining a larger magnetisation between 1 T and the onset of QTM, suggesting slower in-field dynamics for 1 in this range. The hysteresis curves remain marginally open around zero field at 2 and 4 K (with sweep rate of 22 Oe s^−1^ in this region) but without a significant coercive field or remanent magnetisation (Fig. 3). The coordination of the WCA in 1 and 2 significantly increases the magnetic relaxation rate compared to that of the separated ion-pair complex [Dy(Cp^ttt^)_2_][B(C_6_F_5_)_4_] (*T*_H_ = 60 K). However, the hysteresis loops of 1 and 2 are more open at 2 K than for [Dy(Cp^ttt^)_2_(Cl)] which is closed at zero field and only slightly open in fields <1 T,^10,11^ showing the positive effect of the delocalisation of the negative charge in the WCAs compared to chloride. By contrast, the *T*_H_ of the contact-ion pair complex [Dy(Cp^ttt^)(Cp*){Al[OC(CF_3_)_3_]_4_-κ-*F*}] (36 K) is only slightly lower than that of the separated ion-pair complex [Dy(Cp^ttt^)(Cp*)][Al{OC(CF_3_)_3_}_4_] (52 K),^15^ showing that the binding of the WCAs in 1 and 2 is relatively strong in comparison to this literature example; the WCA binding in 1 and 2 has also had a greater effect on the *T*_H_ values of a dysprosocenium cation than previously seen for weakly-bound haloarenes.^21,54^

**Fig. 4:**
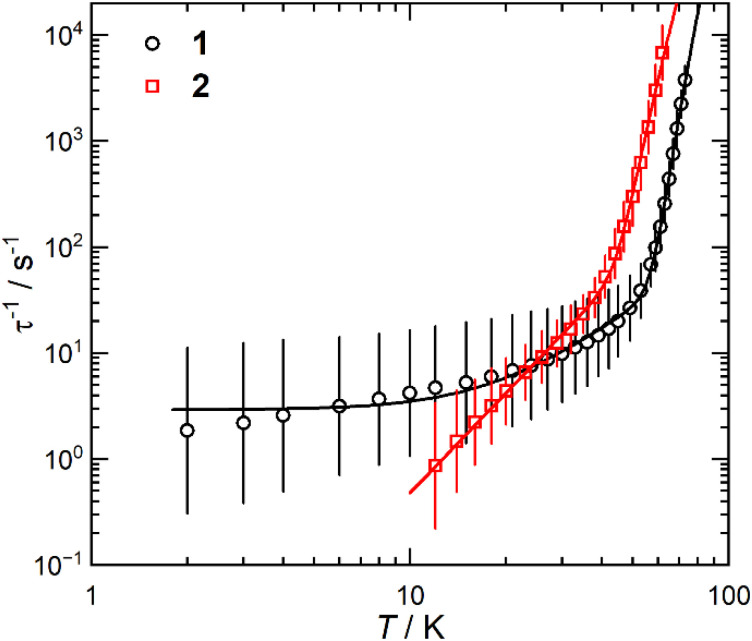
Temperature-dependent magnetic relaxation rates of 1 and 2. Relaxation rates are derived from AC susceptibility data. Error bars represent one standard deviation in the logarithmic distribution of relaxation rates. Lines of best fit are given by eqn (2).

For zero-field AC susceptibility data, peaks are observed between 2–73 K for 1 and 12–59 K for 2; these data fitted well to the generalised Debye model in CC-FIT2^55,56^ and the relaxation profile was extracted (Fig. 4). At low temperatures the relaxation rates of complex 2 lie between the AC and DC timescales and are too fast to be accurately characterised by DC magnetisation decay measurements. Predominantly Orbach relaxation mechanisms are observed at high temperatures until the Raman process begin to dominate at around 50 K for 1 and 40 K for 2. For 1, a plateau in the data <20 K is indicative of QTM starting to dominate, for 2 QTM is not observed within the observable range. The relaxation profiles were fit to a combination of Orbach 
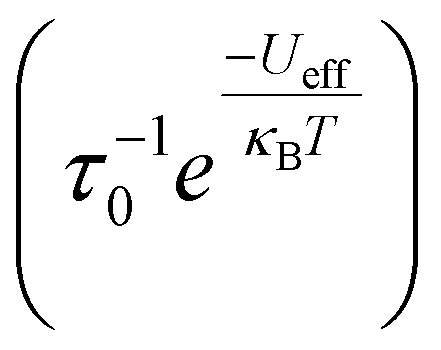
, Raman (*CT*^*n*^) and QTM (τ^−1^_QTM_) processes (1 only);^55,56^ the resultant parameters are compiled in Table 2. The differences in the magnetic properties of 1 [*U*_eff_ = 886(17) cm^−1^] and 2 [*U*_eff_ = 559(18) cm^−1^] are ascribed to the variations in their crystal fields, with longer Dy–Cl and shorter Dy⋯Cp^ttt^_centroid_ distances in 1 contributing to the larger *U*_eff_. By contrast, [Dy(Cp^ttt^)_2_(Cl)] relaxes *via* a Raman process rather than an over-barrier Orbach process, and this slow relaxation could only be observed in an applied DC field,^9^ thus slower QTM in 1 and 2 can be attributed to elongation of the Dy–Cl bonds. The large difference in *U*_eff_ between 1, 2 and [Dy(Cp^ttt^)_2_][B(C_6_F_5_)_4_] (1223(14) cm^−1^);^8^ is in contrast to [Dy(Cp^ttt^)(Cp*)][Al{OC(CF_3_)_3_}_4_] and [Dy(Cp^ttt^)(Cp*){Al[OC(CF_3_)_3_]_4_-κ-*F*}], which exhibit similar *U*_eff_ values upon the coordination of the WCA [1221(25) and 1265(15) cm^−1^, respectively].^15^ It was previously reported than low Raman exponents are characteristic of heavy 4f-metallocenium cations and that this property is disrupted by Cl^−^ coordination;^9^ we find that this is a gradual transition as the value of *n* trends with the strength of the Dy–Cl interaction: [Dy(Cp^ttt^)_2_][B(C_6_F_5_)_4_] ≈ 1 < 2 < [Dy(Cp^ttt^)_2_(Cl)] [*n* = 2.151, 2.3(2), 3.13(9) and 5.3 s^−1^ K^−*n*^, respectively].^8,9^ The differing Raman rates in 1 and 2 are attributed to both crystal field effects and the different low-energy vibrations of the two WCAs;^57^ the coordination of a WCA and subsequent introduction of low-energy vibrations was observed to increase Raman rates in [Dy(Cp^ttt^)(Cp*){Al[OC(CF_3_)_3_]_4_-κ-*F*}].^15^

**Table 2:** Selected magnetic properties of 1 and 2

	1	2
*U*_eff_	886(17) cm^−1^	559(18) cm^−1^
*τ*_0_	10^−11.1(2)^ s	10^−9.4(2)^ s
*C*	10^−2.5(3)^ s^−1^ K^−*n*^	10^−3.45(14)^ s^−1^ K^−*n*^
*n*	2.3(2)	3.13(9)
*τ*_QTM_	10^−0.46(4)^ s	—

**Fig. 5:**
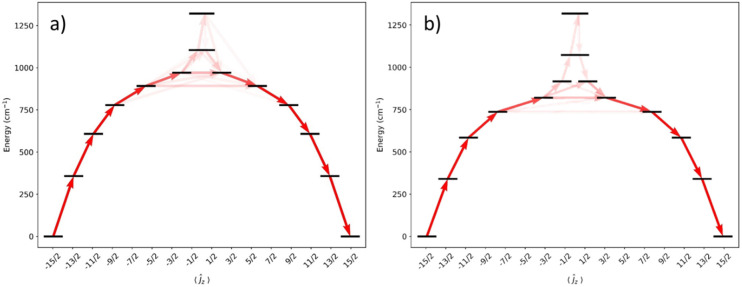
Energy barrier to magnetic relaxation for models of 1 and 2. Electronic states from CASSCF-SO calculations, labelled with their dominant *m*_*J*_ composition in the *J* = 15/2 basis. Arrows represent the Orbach relaxation pathway, where the opacity of the arrows is proportional to the transition probability approximated with the average matrix elements of magnetic moment connecting the states, *γ*_*ij*_ = (1/3)[|<*i*|*μ*_*x*_|*j*>|^2^ + |<*i*|*μ*_*y*_|*j*>|^2^ + |<*i*|*μ*_*z*_|*j*>|^2^], normalised from each departing state and commencing from |–15/2>.

CASSCF-SO calculations were performed on models of 1 and 2 using the metrical parameters obtained from single crystal XRD in OpenMolcas^58^ (see Fig. 5 and ESI Tables S4 and S5†). The calculated *U*_eff_ values [1: 778 cm^−1^; 2: 584 cm^−1^], derived from when the *g*_*x*_ and *g*_*y*_ contributions surpass 0.1, are comparable to measured values proceeding *via* the 3^rd^ excited ±*m*_*J*_ state for 1 and the 2^nd^ excited state for 2; a better match of the calculated and experimental *U*_eff_ values of 1 is for Orbach relaxation to proceed *via* the 4^th^ excited state (892 cm^−1^). The CASSCF calculations do not explain the faster QTM in 1 than 2: ground states for 1 and 2 are dominated by 98% and 97% *m*_*J*_ = ±15/2 respectively, with transverse *g*-values of up to 1–2 × 10^−4^.

## Conclusions

Two dysprosium metallocene complexes have been reported that have equatorially bound WCAs. During the synthetic investigations we found that the *in situ* formation of larger WCAs was not viable by the routes investigated. By a combination of single crystal XRD, magnetic measurements and *ab initio* calculations we find that the WCAs in the two novel complexes herein bind relatively strongly compared to literature examples of WCA- and haloarene-bound dysprosium metallocene complexes but are more weakly coordinating than chloride. The transverse fields introduced using the WCAs herein almost quench magnetic hysteresis entirely, and greatly reduce the effective magnetic barriers to magnetic relaxation, but significant differences are seen between the two complexes. Together, these data provide fresh insights into the sensitivity of dysprosocenium cations to weak equatorial ligand fields.

## Experimental

### Materials and methods

All manipulations were performed in an inert atmosphere (argon) with rigorous exclusion of oxygen and water using Schlenk line and glovebox techniques. Fluorobenzene was dried by stirring with CaH_2_ overnight and was stored over 4 Å molecular sieves. Pentane was dried over a column charged with alumina and stored over potassium mirrors. Anhydrous benzene was purchased and was stored over 4 Å molecular sieves. All solvents were degassed before use. For NMR spectroscopy C_6_D_6_ was dried by refluxing over K, and was vacuum transferred and degassed by three freeze–pump–thaw cycles before use. The reagent AlEt_3_ (1 M in heptane) was purchased from Sigma-Aldrich and used as received. The reagents [Dy(Cp^ttt^)_2_(Cl)],^8,10,11^ HOC(C_6_F_5_)_3_,^59^ PhF–Al{OC(CF_3_)_3_}_3_ ^43^ [Al{OC(C_6_F_5_)_3_}_3_]^45^ were synthesised according to literature procedures. The reagent [Al{OC(C_6_F_5_)_3_}_2_(C_2_H_5_)] was synthesised *via* an adapted literature procedure.^45^^1^H (400 and 500 MHz), ^13^C{^1^H} (126 MHz) and ^19^F (376 MHz) NMR spectra were obtained on a Bruker Avance III 400 or 500 MHz spectrometer at 298 K and were referenced to the solvent used, or to external TMS (^1^H, ^13^C), or C_7_H_5_F_3_/CDCl_3_ (^19^F). ATR-IR spectra were recorded on a Bruker Alpha spectrometer with Platinum-ATR module. Elemental analysis was carried out by Mr Martin Jennings and Mrs Anne Davies at the Microanalytical service, Department of Chemistry, the University of Manchester.

### Synthetic procedures

#### [Dy(Cp^ttt^)_2_{AlCl[OC(CF_3_)_3_]_3_-κ-Cl}] (1)

[Dy(Cp^ttt^)_2_(Cl)] (0.332 g, 0.5 mmol) and PhF–Al{OC(CF_3_)_3_}_3_ (0.442 g, 0.5 mmol) were cooled to 0 °C. Fluorobenzene (10 mL) was added and the reaction mixture was stirred at 0 °C for 4 h. The solvent was removed *in vacuo*, and pentane (40 mL) was added to extract the product. The solvent was removed under vacuum until crystals started to form. The solution was stored at −30 °C and bright yellow crystals formed overnight. The solvent was decanted and residual volatiles were removed under vacuum to afford 1 (0.407 g, 0.3 mmol, 58%). Anal. calcd (%) for C_46_H_58_AlClDyF_27_O_3_: C, 39.55; H, 4.13. Found: C, 38.27; H, 4.33. *μ*_eff_ = 10.63*μ*_B_ (Evans method, C_6_D_6_, 298 K). The paramagnetism of 1 precluded the assignment of ^1^H, ^13^C{^1^H} and ^19^F NMR spectra. FTIR (ATR, microcrystalline): *ν̃* = 2966 (s, C–H stretch), 2908 (w, C–H stretch), 2875 (w, C–H stretch), 1483 (w), 1463 (w), 1398 (w), 1361 (m), 1301 (s), 1260 (s, C–F stretch), 1235 (s), 1217 (s), 1178 (s), 1093 (br. s), 1019 (s), 972 (s), 859 (m), 828 (s), 799 (s), 728 (s), 686 (m), 563 (m), 538 (s), 444 (s).

#### [Dy(Cp^ttt^)_2_{AlCl(C_2_H_5_)[OC(C_6_F_5_)_3_]_2_-κ-Cl}] (2)

Fluorobenzene (10 mL) was added to [Dy(Cp^ttt^)_2_(Cl)] (0.330 g, 0.5 mmol) and [Al{OC(C_6_F_5_)_3_}_2_(C_2_H_5_)] (0.807 g, 0.5 mmol) at room temperature. The yellow reaction mixture was stirred overnight, concentrated under vacuum to *ca*. 2 mL, and yellow crystals formed at room temperature overnight. The solvent was decanted, and residual volatiles removed under vacuum to afford 2 (0.593 g, 0.3 mmol, 60%). Anal. calcd (%) for C_86_H_73_AlClDyF_32_O_2_: C, 52.40; H, 3.73. Found: C, 49.84; H, 3.30. *μ*_eff_ = 10.20*μ*_B_ (Evans method, C_6_D_6_, 298 K). The paramagnetism of 2 precluded the assignment of ^1^H, ^13^C{^1^H} and ^19^F NMR spectra. FTIR (ATR, microcrystalline): *ν̃* = 2963 (m, C–H stretch), 2910 (w, C–H stretch), 2869 (w, C–H stretch), 1650 (s), 1594 (w), 1525 (s), 1490 (s), 1402 (m), 1363 (w), 1303 (s), 1264 (w), 1239 (m), 1215 (s), 1153 (s), 1130 (s), 1112 (s), 1023 (s), 995 (s), 993 (s), 980 (s), 960 (s), 873 (m), 828 (m), 795 (s), 787 (s), 754 (s), 699 (s), 684 (s), 649 (w), 631 (s), 619 (s), 569 (m), 499 (m), 442 (m), 427 (w).

#### [Al(C_2_H_5_){OC(C_6_F_5_)_3_}_2_]

Toluene (100 mL) was added to HOC(C_6_F_5_)_3_ (5.300 g, 10 mmol), the solution was added to triethylaluminium (5 mL, 5 mmol, 1.0 M in heptane) in toluene (50 mL) at −78 °C. The solution was stirred at for 1 h, then warmed to room temperature and stirred for 1 h. The reaction mixture was then stirred at 90 °C for 1 h; gas evolution was observed and the reaction was stopped once this ceased. The colourless solution was concentrated to *ca*. 70 mL under vacuum and stored at −35 °C overnight to yield a white precipitate. The solution was decanted, the product washed with pentane (2 × 30 mL) and volatiles removed *in vacuo* to obtain the product (3.453 g, 3.1 mmol, 62%). Anal. calcd (%) for C_40_H_5_AlF_30_O_2_: C, 43.11; H: 0.45. Found: C, 43.30; H, 0.39. A septet in the ^1^H NMR spectrum at 3.51 ppm (*J* = 2.0 Hz) corresponds to an impurity that we were not able to assign; correlation spectroscopy was inconclusive when we attempted to resolve its identity. However, elemental analysis results obtained were in good agreement with expected values for [Al(C_2_H_5_)(OC(C_6_F_5_)_3_)_2_], so this material was used without further purification. ^1^H NMR (400.13 MHz, C_6_D_6_, 298 K): *δ* = 3.64 (q, 2H, ^3^*J*_HH_ = 7.0 Hz, C*H*_2_CH_3_), 0.84 (t, 3H, ^3^*J*_HH_ = 7.0 Hz, CH_2_C*H*_3_). ^13^C{^1^H} NMR (125.79 MHz, C_6_D_6_, 298 K): *δ* = 146.1–136.5 (m, *C*_6_F_5_), 73.6 (*C*H_2_CH_3_), 14.4 (CH_2_*C*H_3_). ^19^F NMR (376.46 MHz, C_6_D_6_, 298 K): *δ* = 140.8 (d, ^3^*J*_FF_ = 20.0 Hz, C_6_F_5_-*o*), 151.2 (t, ^3^*J*_FF_ = 20.0 Hz, C_6_F_5_-*p*), 161.0 (t, ^3^*J*_FF_ = 20.0 Hz, C_6_F_5_-*m*).

### X-ray crystallography

Crystals of 1 were examined using a Rigaku XtalLAB AFC11 diffractometer with a Hybrid Photon Counting area detector and mirror-monochromated Cu Kα (*λ* = 1.54178 Å) or Mo Kα radiation (*λ* = 0.71073 Å). Crystals of 2 were examined using an Oxford Diffraction Supernova diffractometer, furnished with a CCD area detector and a mirror-monochromated Mo Kα radiation (*λ* = 0.71073 Å). Intensities were integrated from data recorded on 1° frames by *ω* or *φ* rotation. Cell parameters were refined from the observed positions of all strong reflections in each data set. A Gaussian grid face-indexed absorption correction with a beam profile was applied to all structures.^60^ The structures were solved by direct and heavy atom methods using SHELXS or dual-space methods using SHELXT;^61^ the datasets were refined by full-matrix least-squares on all unique *F*^2^ values,^61^ with anisotropic displacement parameters for all non-hydrogen atoms, and with constrained riding hydrogen geometries; *U*_iso_(H) was set at 1.2 (1.5 for methyl groups) times *U*_eq_ of the parent atom. The largest features in final difference syntheses were close to heavy atoms and were of no chemical significance. CrysAlisPro^60^ was used for control and integration, and SHELXL^61,62^ was employed through OLEX2^63^ for structure solution and refinement. ORTEP-3^64^ and POV-Ray^65^ were employed for molecular graphics.

### Magnetic measurements

Magnetic measurements were made using a Quantum Design MPMS3 superconducting quantum interference device (SQUID) magnetometer. All samples were prepared in the same manner. Samples were crushed with a mortar and pestle under an inert atmosphere, and then loaded into a borosilicate glass NMR tube along with eicosane, which was then evacuated and flame-sealed to a length of *ca*. 5 cm. The eicosane was melted by heating the tube gently with a low-power heat gun in order to immobilise the crystallites. The NMR tube was then mounted in the centre of a drinking straw using friction by wrapping it with Kapton tape, and the straw was then fixed to the end of the sample rod. For 1 25.6 mg of sample was prepared with 15.4 mg of eicosane, for 2 21.0 mg sample with 12.9 mg eicosane. The measurements were corrected for the diamagnetism of the straw, borosilicate tube and eicosane using calibrated blanks, and for the intrinsic diamagnetism of the sample estimated as the molecular weight (g mol^−1^) multiplied by −0.5 × 10^−6^ cm^3^ K mol^−1^. The moment was also corrected for the shape of the sample (divided by 1.022 for 1 and 1.029 for 2), calculated with the Quantum Design Geometry Simulator assuming a perfectly cylindrical sample shape with height 3.79 mm and diameter 4.06 mm (1) or height 3.31 mm and diameter 4.05 mm (2).

All DC magnetic measurements were performed in DC scan mode with a scan length of 40 mm and a scan time of 6 s. The equilibrium magnetic susceptibility was measured under 0.1 T field, on cooling in temperature settle mode. For 1 the sweep rates were 5 K min^−1^ from 300–100 K, 2 K min^−1^ from 100–10 K and 1 K min^−1^ from 10–1.8 K, with addition waits of 2, 4, 10 and 18 minutes at 3.0, 2.5, 2.1 and 1.8 K, respectively. For 2 the sweep rates were 5 K min^−1^ from 300–100 K, 2 K min^−1^ from 100–50 K and 1 K min^−1^ from 50–1.8 K. Equilibrium magnetisation *vs*. field measurements held the temperature and field stable for 10 min (2 K) or 5 min (4 K) before each measurement. Hysteresis measurements were performed in continuous sweep mode between ±7 T on a sample that had been magnetised at 7 T. The sweep rates were 22 Oe s^−1^ for |*H*| < 1 T, 54 Oe s^−1^ for 1 < |*H*| < 2 T, and 91 Oe s^−1^ for 2 < |*H*| < 7 T.

Alternating frequency (AC) susceptibility measurements were recorded for 8 frequencies per decade between 0.1–1000 Hz with a 2 Oe oscillating field. Averages were performed for 2 s or for 10 cycles, whichever was longer. Slow thermalisation was observed below 40 K for 1 (much slower at 21 K and below) and below 20 K for 2, so after changing temperature the ac susceptibility was monitored as a function of time to ensure complete thermalisation before recording the frequency dependence of the ac susceptibility.

AC data were fit to the Generalised Debye model (eqn (1)) in CC-FIT2 to extract relaxation rates and distributions.^55,56^1
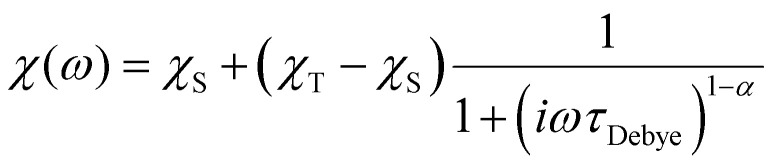
where *τ*_Debye_^−1^ is the relaxation rate, *ω* is the angular frequency of the AC field and *χ*_T_ and *χ*_S_ are the isothermal and adiabatic susceptibilities, respectively. There is good agreement between the model and the data (Fig. S17–S22†). The resultant parameters are shown in Tables S2–S3.†

The temperature-dependence of the rates of 1 and 2 in zero-field was fit to:2
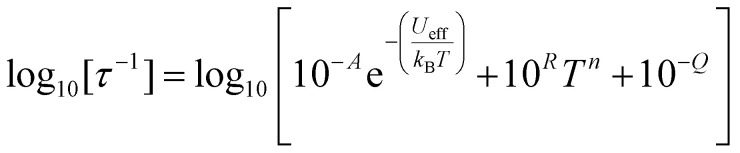
where 10^−*A*^ s^−1^ (*τ*_0_^−1^) is the Orbach prefactor, *U*_eff_ is the effective energy barrier for the Orbach process, 10^*R*^ s^−1^ K^−*n*^ (*C*) and *n* are phenomenological parameters that describe the Raman process, and 10^−*Q*^ s^−1^ (*τ*_QTM_^−1^) is the quantum tunnelling of magnetisation (QTM) rate; for 2 the QTM rate is omitted as rates were determined only for 12 K and above and no plateau was observed in this region.

### CASSCF-SO calculations

OpenMolcas^58^ was used to perform CASSCF-SO calculations on 1 and 2 to determine the electronic structure. The molecular geometries from the single crystal XRD structures were used with no optimisation. Electron integrals were performed in the SEWARD module using basis sets from ANO-RCC library^66–69^ with VTZP quality for the Dy atom, VDZP quality for the cyclopentadienyl C atoms and the fluorobenzene F atoms, and VDZ quality for all remaining atoms, employing the second-order DKH Hamiltonian for scalar relativistic effects. Resolution of identity Cholesky decomposition (RICD) of the two-electron integrals with atomic compact Cholesky decomposition (acCD) auxiliary basis sets was employed to reduce computational demand.^70^ The molecular orbitals (MOs) were optimised in state-averaged CASSCF (SA-CASSCF) calculations in the RASSCF module, where the active space was defined by the nine electrons in the seven 4f orbitals of Dy(iii). A SA-CASSCF calculation was performed for the lowest 18 sextets, where these states were then mixed by spin orbit coupling in the RASSI module. SINGLE_ANISO was used to decompose the resulting spin–orbit wave functions into the CF Hamiltonian formalism.^71^

## Author contributions

S.C.C. and D.P.M. provided the original concept. S.C.C. synthesised and characterised the complexes. S.C.C. collected and finalised the SCXRD data. S.C.C. performed *ab initio* calculations. G.K.G. collected the magnetic data. G.K.G. and S.C.C. interpreted the magnetic data. G.K.G. supervised the magnetism and calculations components. D.P.M. supervised the synthetic component and directed the research. S.C.C., G.K.G. and D.P.M. wrote the manuscript.

## Data availability

ESI† is available in the online version of the paper. Correspondence and requests for materials should be directed to G.K.G. and D.P.M. Crystallographic data for the structures reported in this Article have been deposited at the Cambridge Crystallographic Data Centre, under deposition numbers CCDC 2386076 (1), 2386077 (2).† Raw research data files supporting this publication are available from Figshare at https://doi.org/10.6084/m9.figshare.27091723. Apart from the data sets mentioned, all other data supporting the findings of this study are available within the Article and ESI.†

## Conflicts of interest

There are no conflicts to declare.

## Supplementary Material

DT-054-D4DT02713B-s001

DT-054-D4DT02713B-s002
