# Supplementary material for: Influence of weakly coordinating anions binding to the hexa-tert-butyl dysprosocenium cation
Source: Dalton Trans. 2024 Nov 5;54(1):198–206. doi: 10.1039/d4dt02713b (PMC11563204; doi:10.1039/d4dt02713b)
Supplement: DT-054-D4DT02713B-s001 [file DT-054-D4DT02713B-s001.pdf]

Supplementary Information (SI) for Dalton Transactions.
This journal is © The Royal Society of Chemistry 2024

Influence of weakly coordinating anions binding to the hexa-*tert*-butyl dysprosocenium cation

Sophie C. Corner,[a] Gemma K. Gransbury,[a] and David P. Mills[a]**

[a]Department of Chemistry, The University of Manchester, Oxford Road, Manchester, M13 9PL, U.K.

Email: david.mills@manchester.ac.uk, gemmagransbury@gmail.com.

Contents

1. **Infrared Spectroscopy** S2
2. **NMR Spectroscopy** S3
3. **X-ray Crystallography** S7
4. **Magnetic Measurements** S10
5. **CASSCF-SO Calculations** S23

1. Infrared Spectroscopy

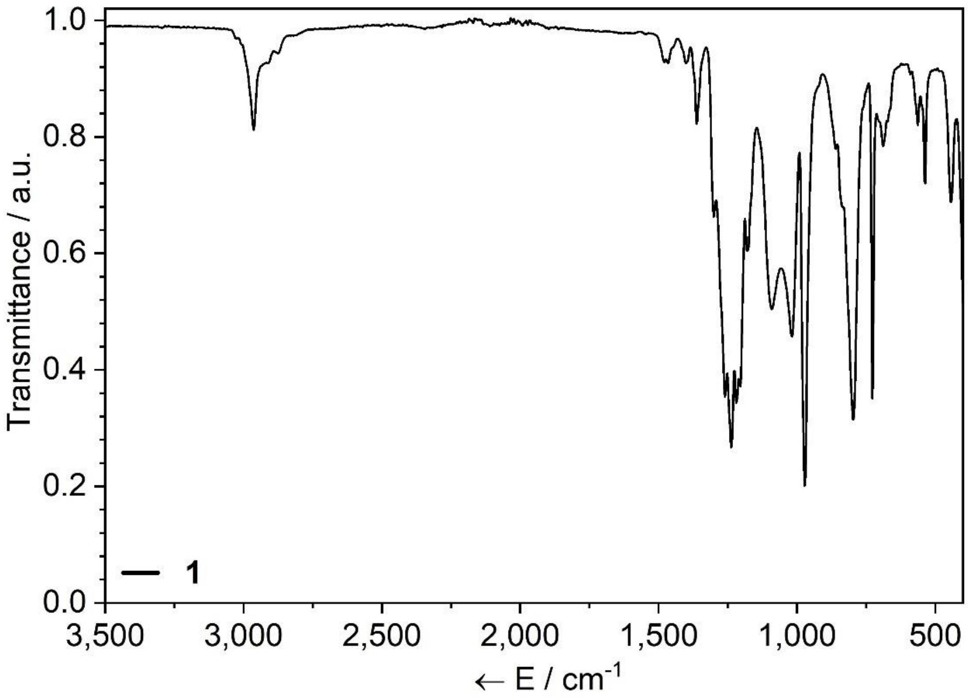

Figure S1. ATR-IR spectrum of **1**, recorded as a microcrystalline powder.

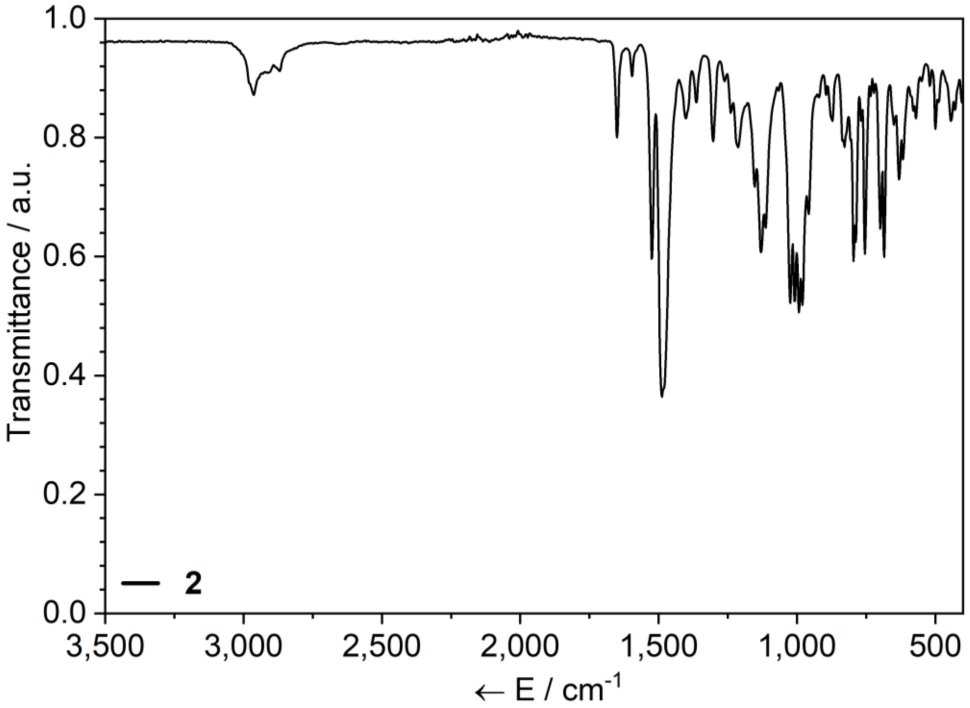

Figure S2. ATR-IR spectrum of **2**, recorded as a microcrystalline powder.

2. NMR Spectroscopy

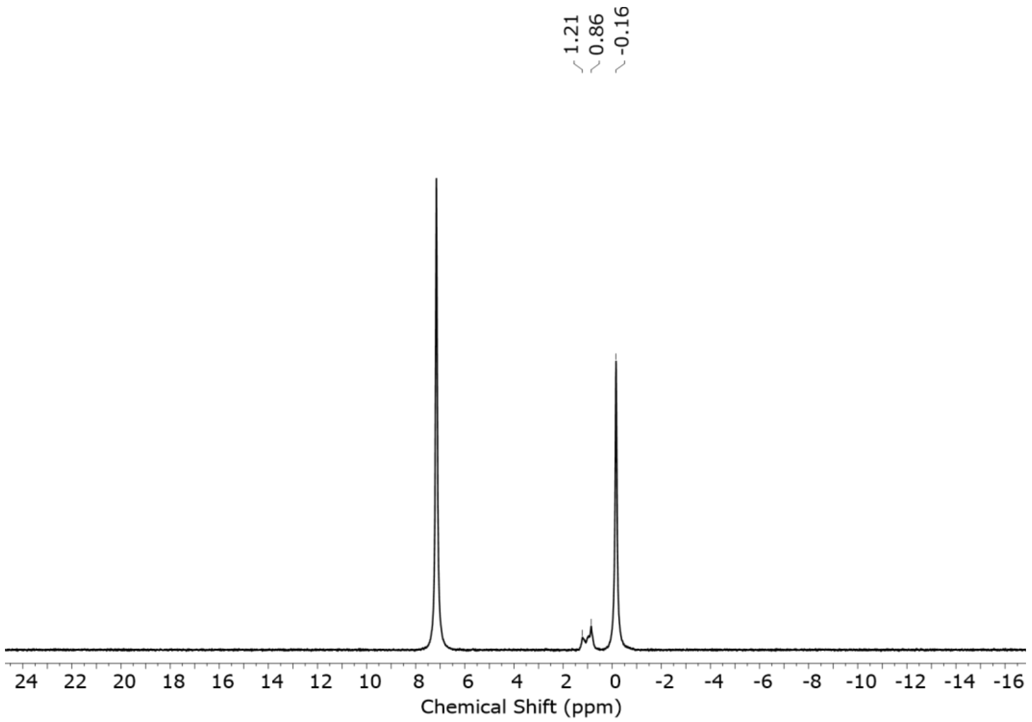

Figure S3. ^{1}H NMR spectrum of **1** (400 MHz) in C_6H_5F; full spectral range 200 to –200 ppm.

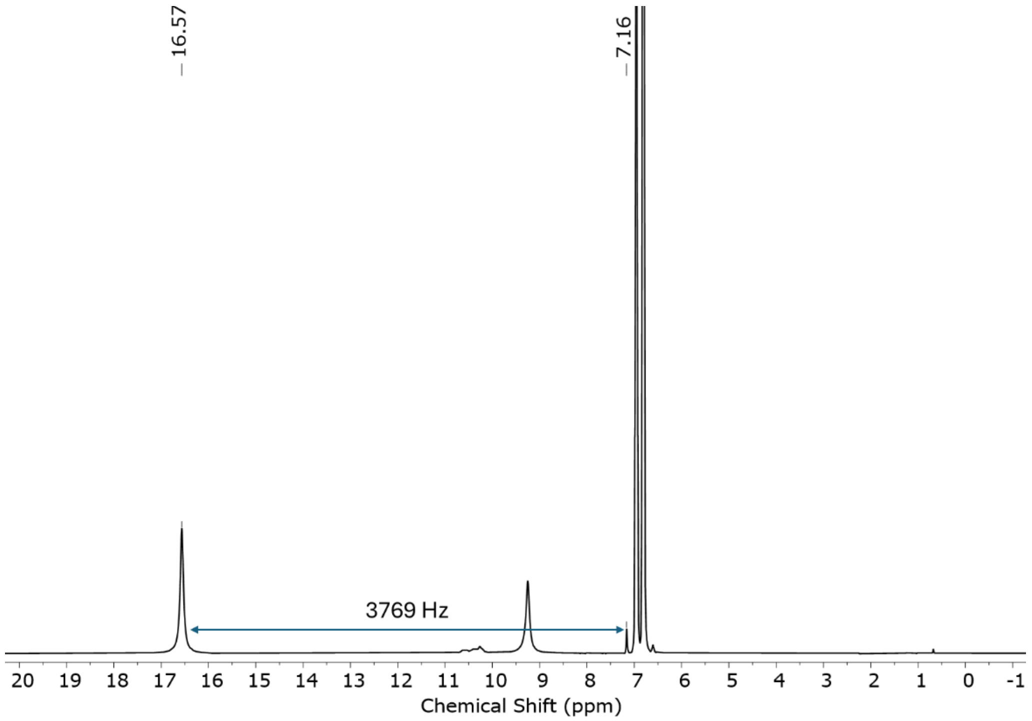

Figure S4. ^{1}H NMR spectrum of **1** (400 MHz) in C_6H_5F with a C_6D_6/C_6H_5F insert; full spectral range 200 to –200 ppm.

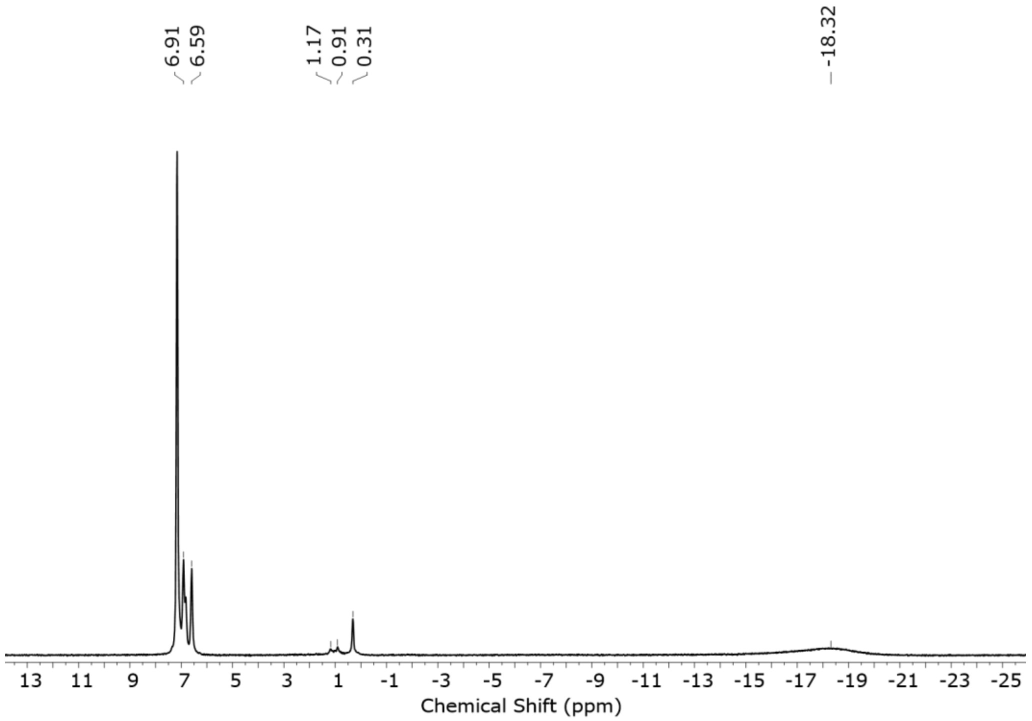

Figure S5. ^{1}H NMR spectrum of **2** (400 MHz) in C_6H_5F; full spectral range 200 to –200 ppm.

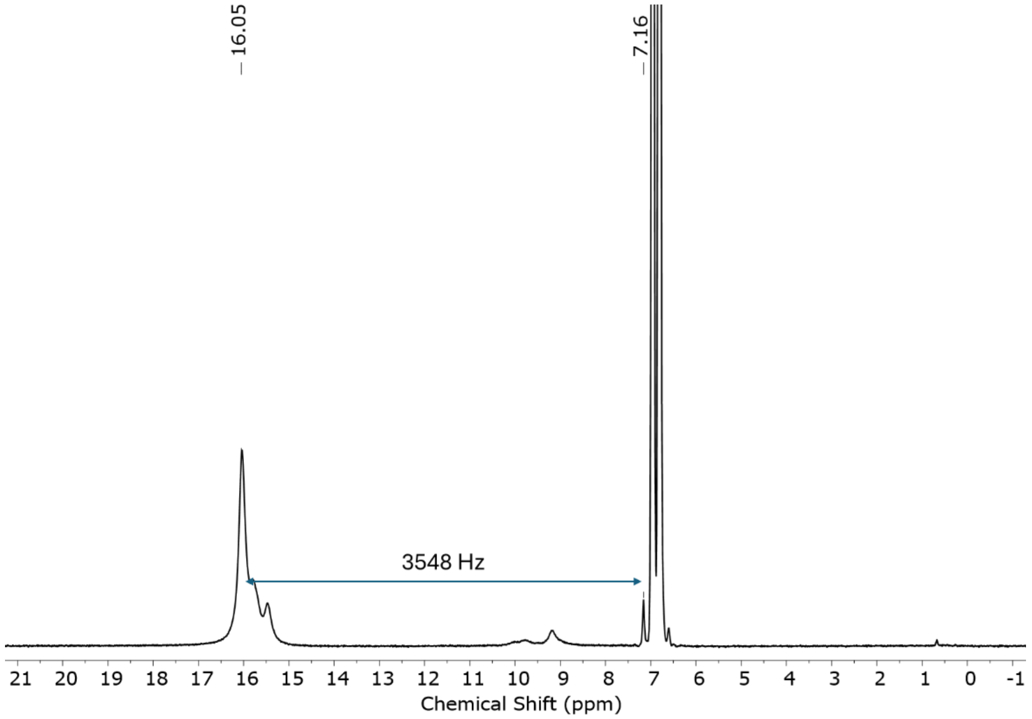

Figure S6. ^{1}H NMR spectrum of **2** (400 MHz) in C_6H_5F with a C_6D_6/C_6H_5F insert; full spectral range 200 to –200 ppm.

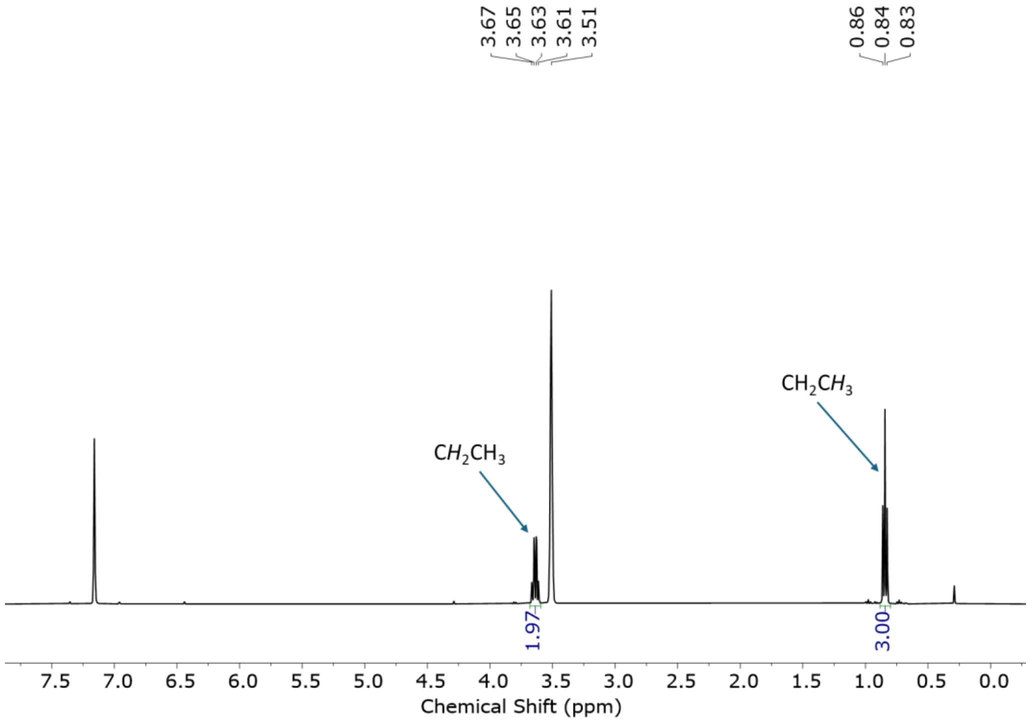

Figure S7. ^{1}H NMR spectrum of $[Al(C_2H_5)\{OC(C_6F_5)_3\}_2]$ (400 MHz) in C_6D_6.

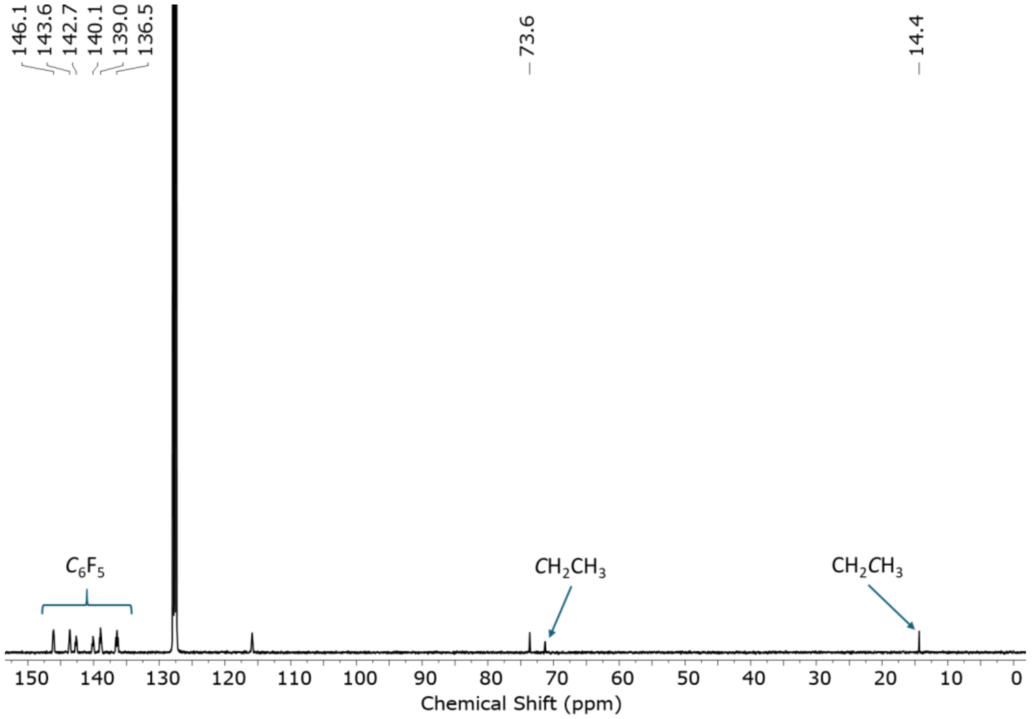

Figure S8. $^{13}C\{^{1}H\}$ NMR spectrum of $[Al(C_2H_5)\{OC(C_6F_5)_3\}_2]$ (126 MHz) in C_6D_6.

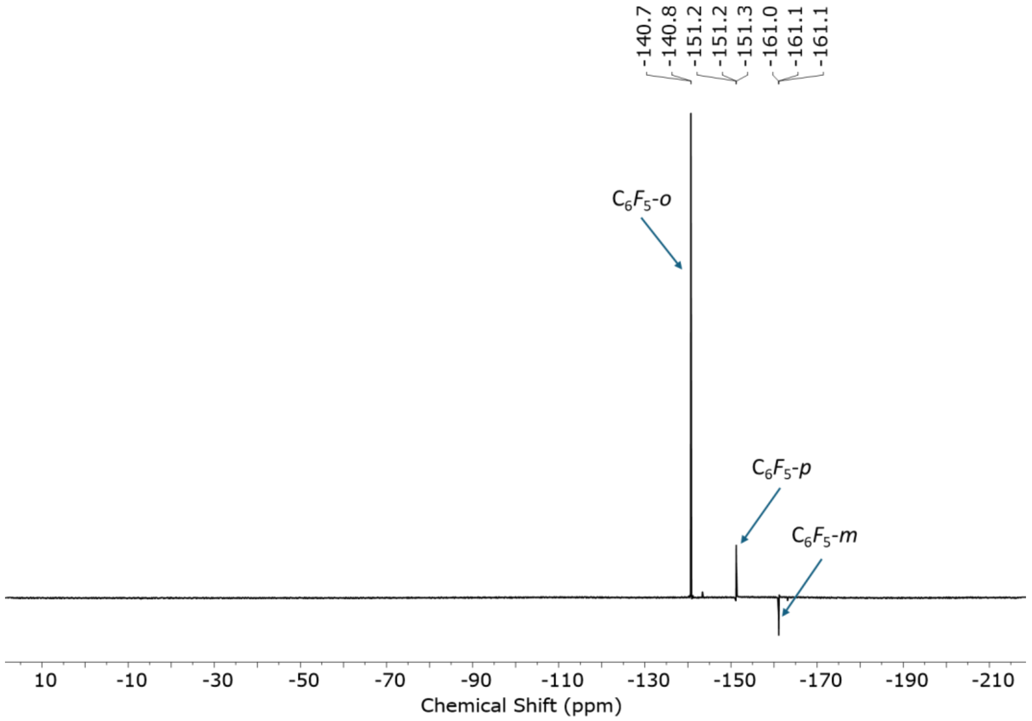

Figure S9. ^{19}F NMR spectrum of $[Al(C_2H_5)\{OC(C_6F_5)_3\}_2]$ (376 MHz) in in C_6D_6.

3. X-ray Crystallography

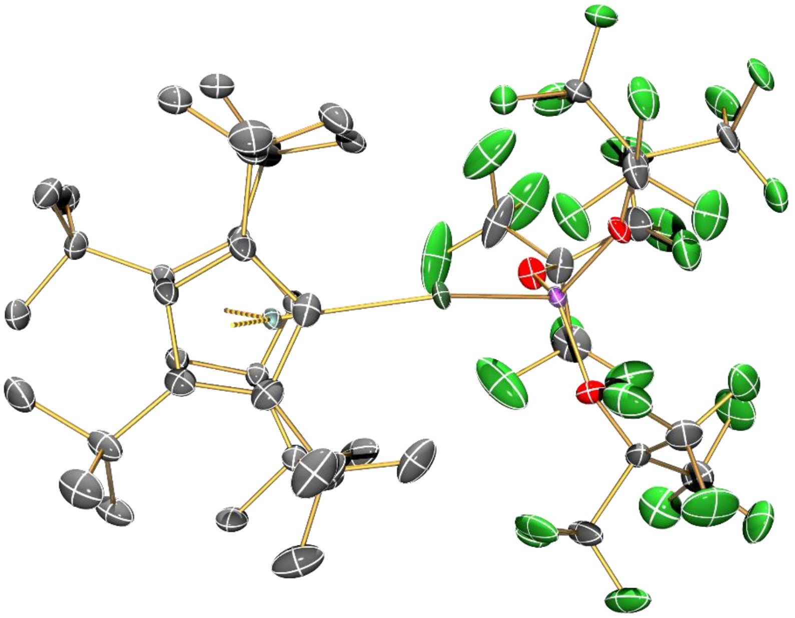

Figure S10. Top view of the single crystal XRD structure of **1** (Dy: cyan, C: grey, Al: purple, F: green, O: red). Displacement ellipsoids set at 30% probability levels; hydrogen atoms have been omitted for clarity.

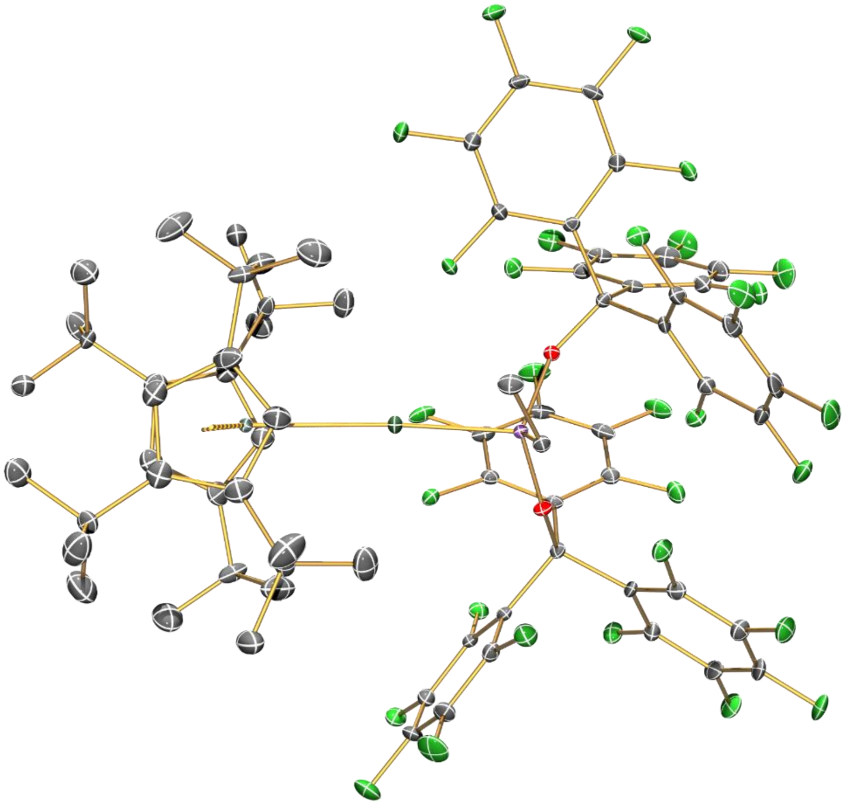

Figure S11. Top view of the single crystal XRD structure of **2** (Dy: cyan, C: grey, Al: purple, F: green, O: red). Displacement ellipsoids set at 30% probability levels; hydrogen atoms and the lattice fluorobenzene molecules have been omitted for clarity.

Table S1. Crystallographic data for **1** and **2**.

	**1**	**2**
Formula	$C_{46}H_{58}AlClDyF_{27}O_3$	$C_{86}H_{73}AlClDyF_{32}O_2$
molecular mass, g mol^{-1}	1396.85	1971.37
cryst size, mm	0.236 × 0.191 × 0.103	0.464 × 0.249 × 0.167
cryst syst	orthorhombic	triclinic
space group	$Pca2_1$	$P\bar{1}$
collection temperature, K	99.97(10)	99.97(10)
a, Å	21.2423(2)	12.3345(3)
b, Å	14.24450(10)	16.0941(5)
c, Å	18.1335(2)	21.5697(7)
α, °	90	72.902(3)
β, °	90	78.936(2)
γ, °	90	85.763(2)
V, $Å^3$	5486.94(9)	4015.9(2)
Z	4	2
ρ_{calcd}, g cm^{-3}	1.691	1.630
μ, mm^{-1}	9.090	1.098
no. of reflections made	38414	36237
no. of unique reflns, R_{int}	10820, 0.0303	18504, 0.0455
no. of reflns with $F^2 > 2\sigma(F^2)$	10124	15087
transmn coeff range	0.524–1.000	0.609–1.000
R, R_w^a ($F^2 > 2\sigma(F^2)$)	0.0451, 0.1287	0.0627, 0.1516
R, R_w^a (all data)	0.0480, 0.1319	0.0807, 0.1623
S^a	1.007	1.017
parameters, restraints	1101, 3151	1191, 1674
max., min. diff map, e $Å^{-3}$	0.597, –0.742	2.437, –1.094

[a] Conventional $R = \Sigma||F_o| - |F_c||/\Sigma|F_o|$; $R_w = [\Sigma w(F_o^2 - F_c^2)^2/\Sigma w(F_o^2)^2]^{1/2}$; $S = [\Sigma w(F_o^2 - F_c^2)^2/\text{no. data} - \text{no. params})]^{1/2}$ for all data.

4. Magnetic Measurements

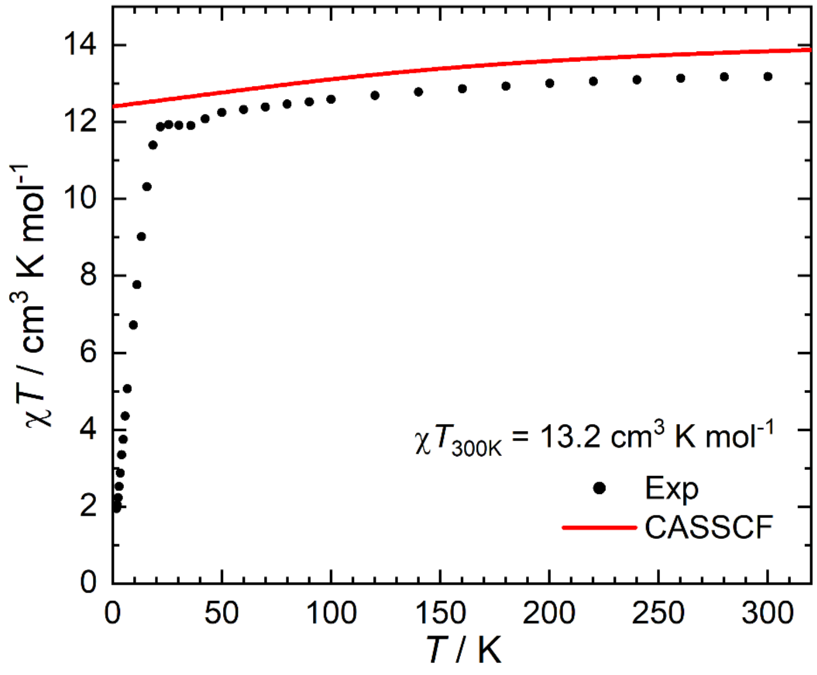

Figure S12. Temperature dependence of the molar magnetic susceptibility (χT) product for powdered **1** measured under a 0.1 T applied magnetic field (black circles) and CASSCF calculated curve (red line).

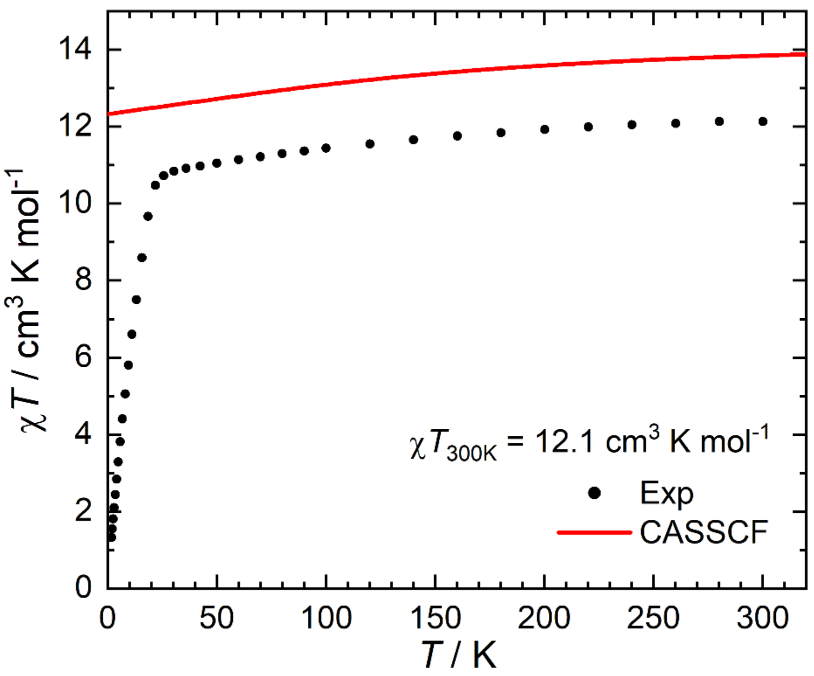

Figure S13. Temperature dependence of the molar magnetic susceptibility (χT) product for powdered **2** measured under a 0.1 T applied magnetic field (black circles) and CASSCF calculated curve (red line).

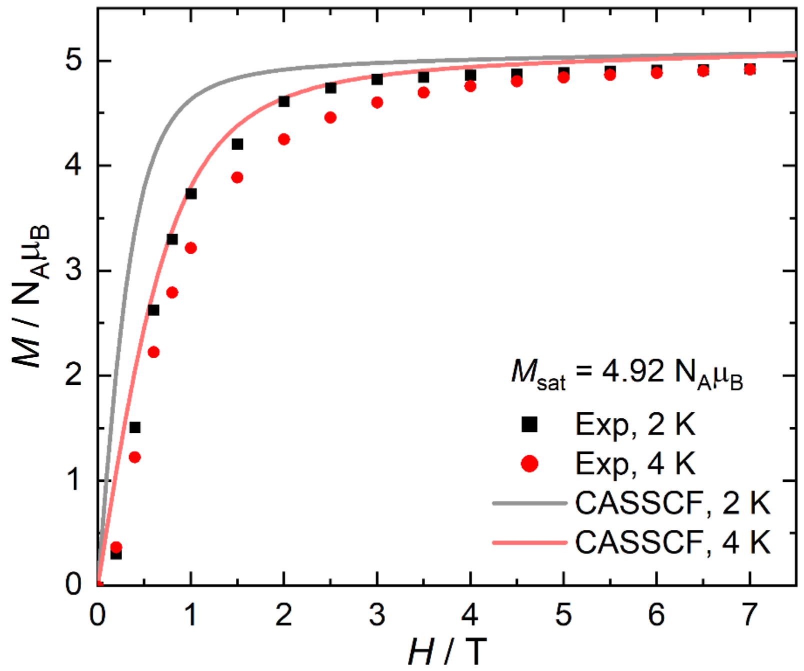

Figure S14. Isothermal magnetisation *versus* field for **1** at 2 (black squares) and 4 K (red circles), with CASSCF calculated curves (solid lines).

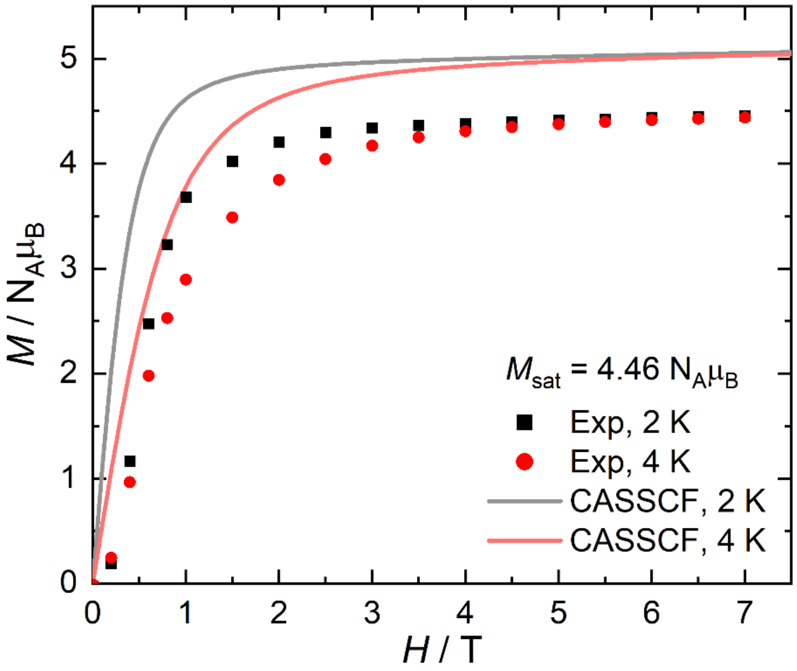

Figure S15. Isothermal magnetisation *versus* field for **2** at 2 (black squares) and 4 K (red circles), with CASSCF calculated curves (solid lines).

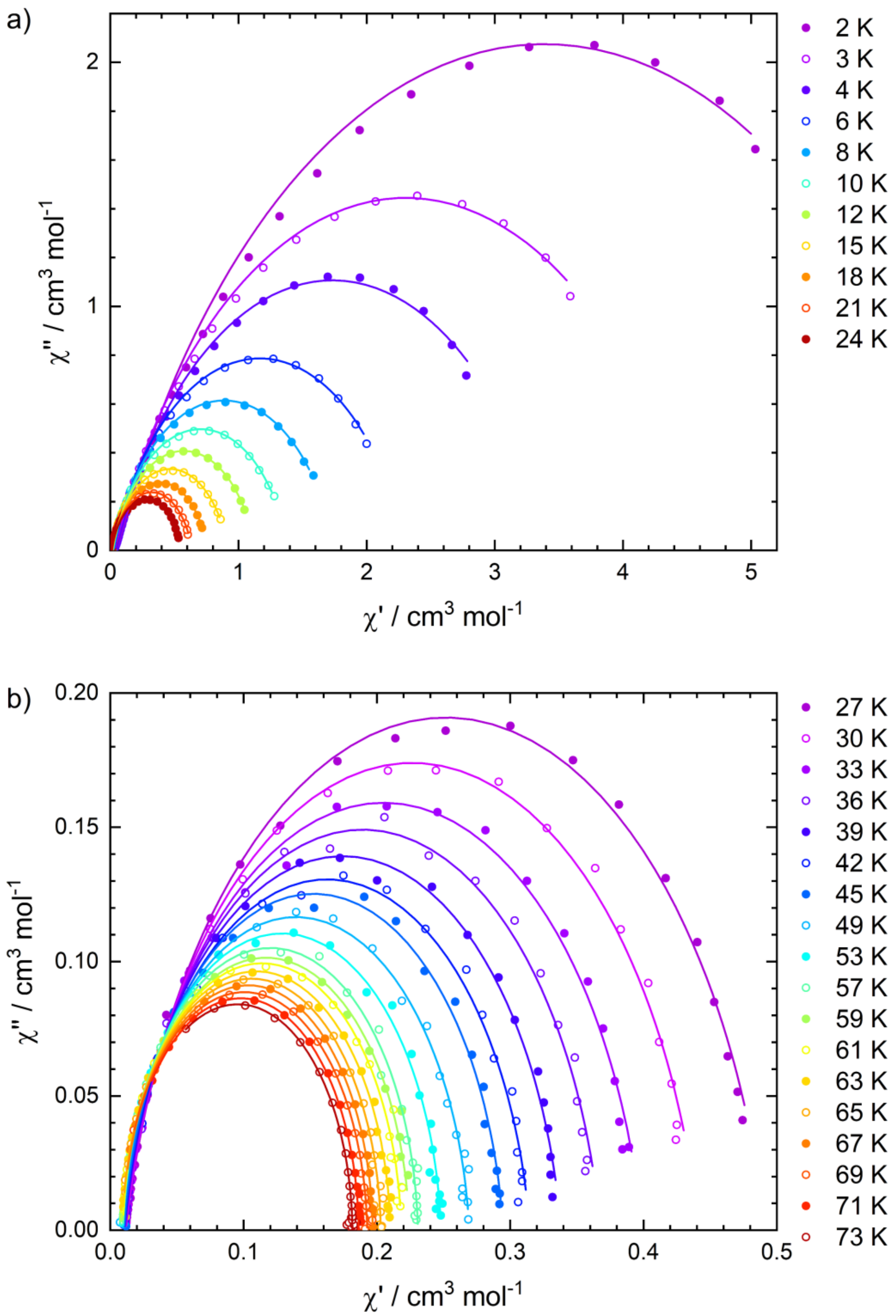

Figure S16. Cole-Cole plot for **1** at a) 2–24 K and b) 27–73 K. Solid lines are fits to the generalised Debye model, giving $0.014 \leq \alpha \leq 0.29$.

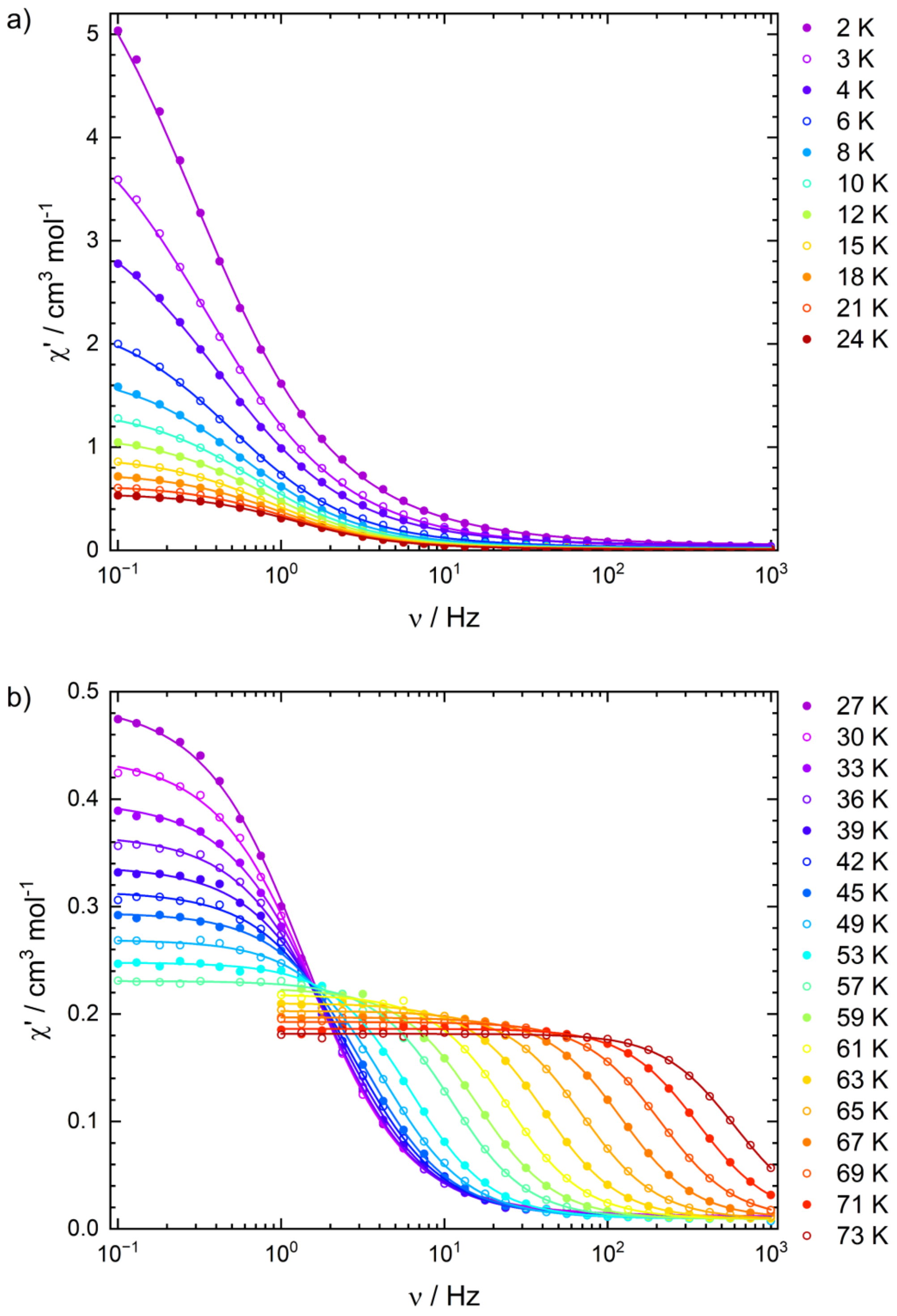

Figure S17. In-phase ac susceptibility (χ′) of **1** in a zero field at a) 2–24 K and b) 27–73 K. Solid lines are fits to the generalised Debye model, giving $0.014 \leq \alpha \leq 0.29$.

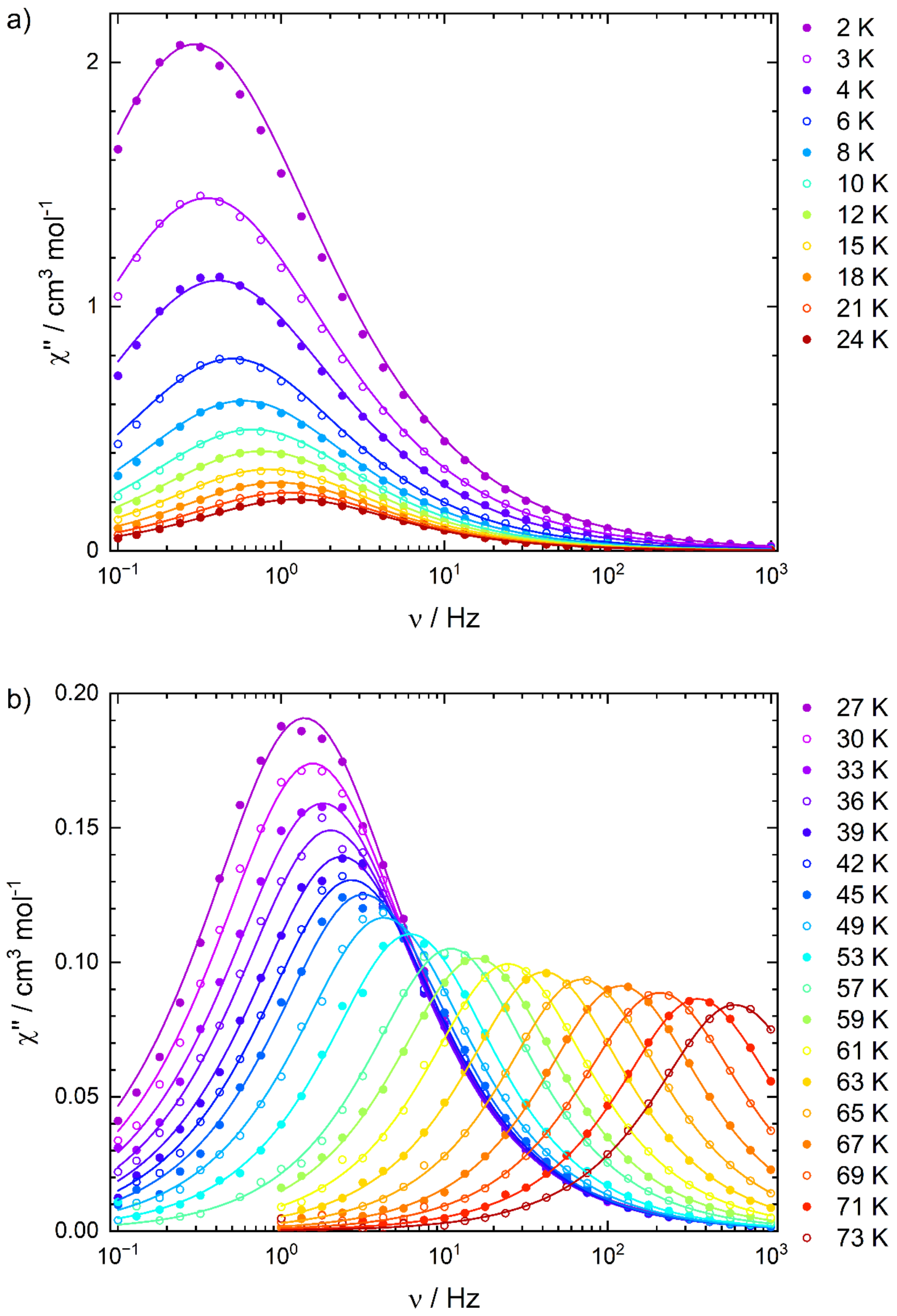

Figure S18. Out-of-phase ac susceptibility (χ'') of **1** in a zero field at a) 2–24 K and b) 27–73 K. Solid lines are fits to the generalised Debye model, giving $0.014 \le \alpha \le 0.29$.

Table S2. Best fit parameters to the generalised Debye model for **1**.

T	τ_{debye}	$\tau_{\mathrm{Debye}}^{\mathrm{err}}$	χ_S	χ_S^{err}	χ_T	χ_T^{err}	α	α^{err}	$\langle \ln \tau \rangle$	$\sigma_{\langle \ln \tau \rangle}$
(K)	(s)		(cm^3/mol)		(cm^3/mol)				ln (s)	
2	5.38E–1	1.02E–2	5.10E–2	8.64E–3	6.72	5.42E–2	2.92E–1	4.81E–3	–0.621	1.807
3	4.55E–1	6.06E–3	2.90E–2	4.80E–3	4.58	2.63E–2	2.79E–1	3.72E–3	–0.788	1.745
4	3.88E–1	4.70E–3	4.77E–2	3.78E–3	3.42	1.81E–2	2.60E–1	3.77E–3	–0.947	1.649
6	3.16E–1	3.66E–3	2.61E–2	2.97E–3	2.30	1.20E–2	2.30E–1	4.14E–3	–1.151	1.504
8	2.72E–1	2.79E–3	2.25E–2	2.25E–3	1.75	8.11E–3	2.14E–1	3.96E–3	–1.302	1.429
10	2.37E–1	1.95E–3	1.95E–2	1.57E–3	1.39	5.13E–3	2.02E–1	3.35E–3	–1.439	1.370
12	2.12E–1	1.36E–3	1.64E–2	1.06E–3	1.13	3.21E–3	1.96E–1	2.70E–3	–1.553	1.343
15	1.90E–1	1.11E–3	1.40E–2	8.26E–4	9.19E–1	2.35E–3	1.91E–1	2.53E–3	–1.663	1.319
18	1.67E–1	1.17E–3	1.33E–2	8.69E–4	7.57E–1	2.28E–3	1.80E–1	3.14E–3	–1.789	1.264
21	1.46E–1	1.07E–3	1.25E–2	8.07E–4	6.36E–1	1.96E–3	1.69E–1	3.38E–3	–1.924	1.216
24	1.30E–1	8.13E–4	1.19E–2	6.32E–4	5.55E–1	1.44E–3	1.61E–1	2.97E–3	–2.039	1.176
27	1.15E–1	9.53E–4	1.16E–2	7.93E–4	4.92E–1	1.68E–3	1.44E–1	4.10E–3	–2.166	1.097
30	1.02E–1	7.19E–4	1.15E–2	6.32E–4	4.42E–1	1.26E–3	1.34E–1	3.57E–3	–2.283	1.047
33	8.83E–2	6.52E–4	1.10E–2	6.25E–4	3.99E–1	1.16E–3	1.26E–1	3.80E–3	–2.428	1.007
36	7.93E–2	5.29E–4	1.09E–2	5.41E–4	3.68E–1	9.57E–4	1.13E–1	3.52E–3	–2.535	0.947
39	6.79E–2	5.54E–4	1.06E–2	6.35E–4	3.38E–1	1.05E–3	1.03E–1	4.37E–3	–2.689	0.893
42	5.90E–2	4.48E–4	1.05E–2	5.69E–4	3.15E–1	8.90E–4	9.68E–2	4.12E–3	–2.831	0.862
45	4.99E–2	3.64E–4	1.04E–2	5.38E–4	2.95E–1	7.86E–4	8.05E–2	4.05E–3	–2.997	0.775
49	3.76E–2	2.24E–4	9.31E–3	4.28E–4	2.69E–1	5.64E–4	6.91E–2	3.37E–3	–3.282	0.712
53	2.58E–2	1.71E–4	9.39E–3	4.75E–4	2.48E–1	5.51E–4	4.90E–2	3.85E–3	–3.658	0.590

57	1.46E–2	1.09E–4	9.52E–3	5.50E–4	2.31E–1	5.58E–4	3.24E–2	4.45E–3	–4.227	0.473
59	1.01E–2	6.28E–5	8.90E–3	4.48E–4	2.24E–1	6.11E–4	3.83E–2	3.87E–3	–4.597	0.517
61	6.46E–3	5.30E–5	9.30E–3	6.35E–4	2.18E–1	7.08E–4	3.17E–2	5.14E–3	–5.042	0.468
63	3.88E–3	1.41E–5	8.83E–3	3.05E–4	2.10E–1	2.72E–4	2.73E–2	2.29E–3	–5.552	0.432
65	2.27E–3	1.50E–5	9.00E–3	6.08E–4	2.03E–1	4.28E–4	2.18E–2	4.20E–3	–6.087	0.385
67	1.32E–3	6.81E–6	9.68E–3	5.31E–4	1.97E–1	2.87E–4	1.63E–2	3.30E–3	–6.632	0.332
69	7.63E–4	6.15E–6	8.47E–3	9.32E–4	1.93E–1	3.79E–4	2.42E–2	4.98E–3	–7.178	0.406
71	4.47E–4	4.36E–6	9.81E–3	1.24E–3	1.86E–1	3.31E–4	1.35E–2	5.61E–3	–7.714	0.301
73	2.65E–4	4.12E–6	9.49E–3	2.05E–3	1.82E–1	3.39E–4	1.42E–2	7.32E–3	–8.237	0.309

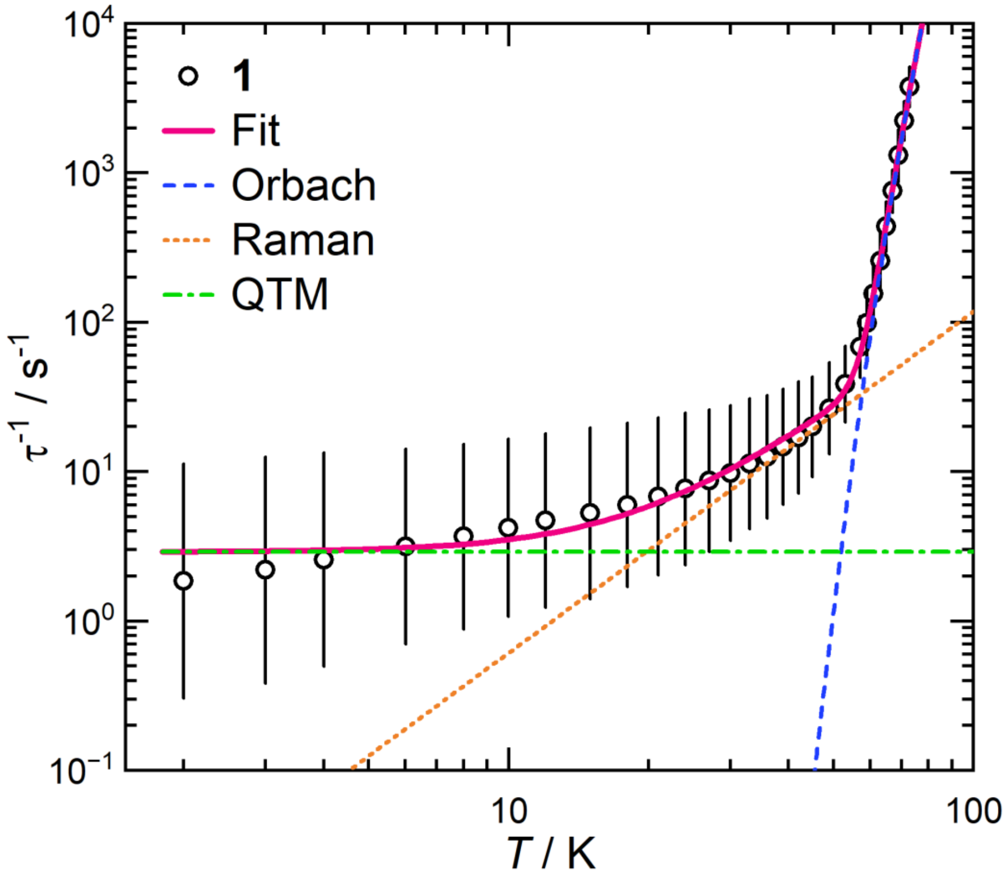

Figure S19. Relaxation profile for **1** showing contributions from Orbach, Raman and QTM processes.

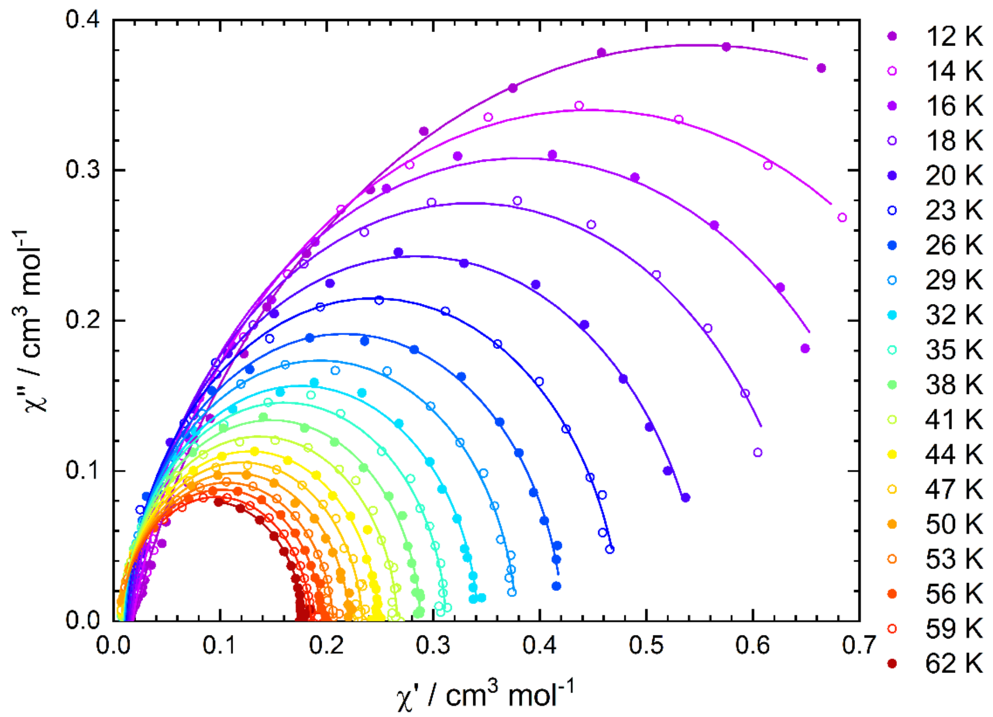

Figure S20. Cole-Cole plot for **2** at 12–62 K. Solid lines are fits to the generalised Debye model, giving $0.025 \leq \alpha \leq 0.20$.

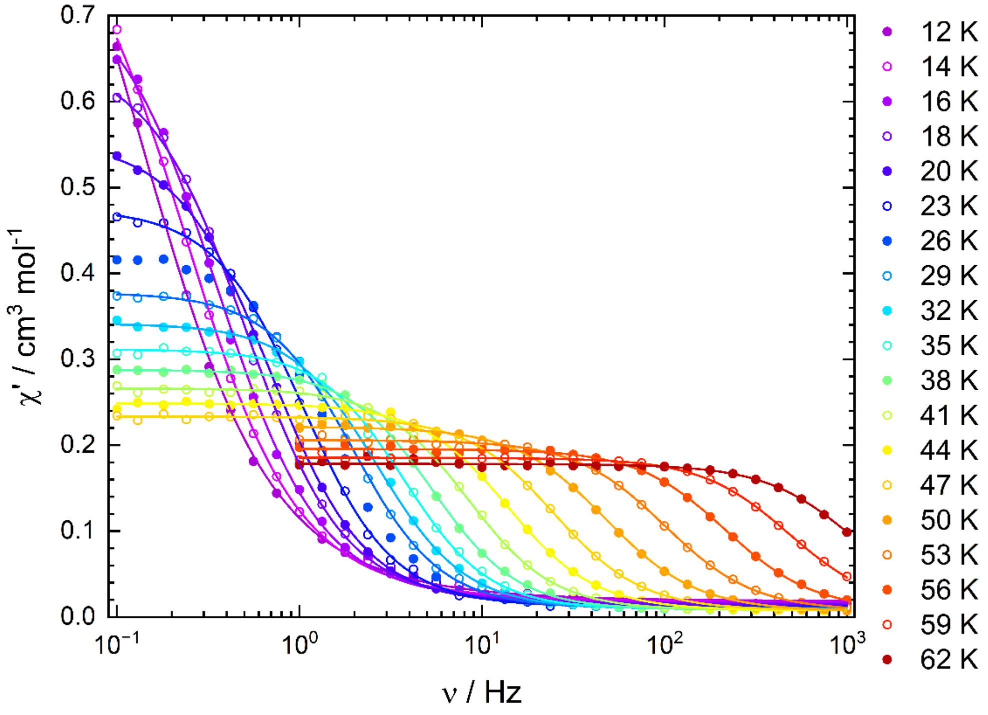

Figure S21. In-phase (χ′) ac susceptibility of **2** in a zero field at 12–62 K. Solid lines are fits to the generalised Debye model, giving $0.025 \leq \alpha \leq 0.20$.

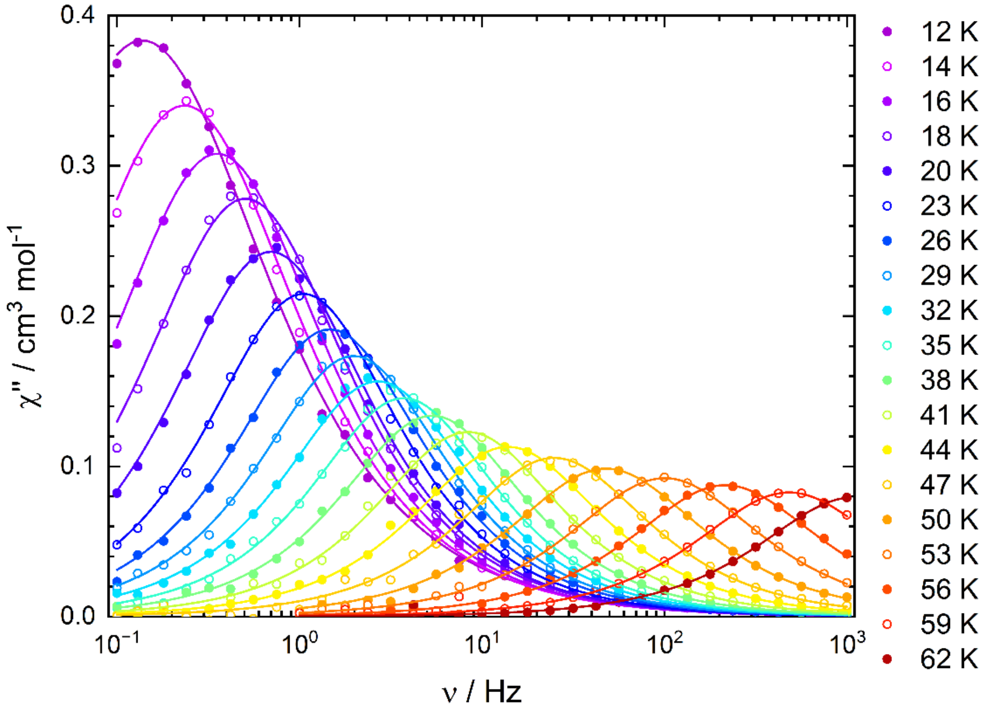

Figure S22. Out-of-phase (χ″) ac susceptibilities of **2** in a zero field at 12–62 K. Solid lines are fits to the generalised Debye model, giving $0.025 \leq \alpha \leq 0.20$.

Table S3. Best fit parameters to the generalised Debye model for **2**.

T	τ_{debye}	$\tau_{\mathrm{Debye}}^{\mathrm{err}}$	χ_{S}	$\chi_{\mathrm{S}}^{\mathrm{err}}$	χ_{T}	$\chi_{\mathrm{T}}^{\mathrm{err}}$	α	α^{err}	$\langle \ln \tau \rangle$	$\sigma_{\langle \ln \tau \rangle}$
(K)	(s)		(cm^3/mol)		(cm^3/mol)				ln (s)	
12	1.15	2.32E–2	1.86E–2	1.01E–3	1.08	1.14E–2	2.02E–1	4.97E–3	0.142	1.370
14	6.78E–1	8.18E–3	1.78E–2	9.27E–4	8.76E–1	5.93E–3	1.46E–1	4.42E–3	–0.389	1.106
16	4.49E–1	4.39E–3	1.63E–2	9.49E–4	7.50E–1	4.17E–3	1.10E–1	4.50E–3	–0.801	0.929
18	3.14E–1	2.82E–3	1.48E–2	9.59E–4	6.56E–1	3.24E–3	8.96E–2	4.64E–3	–1.159	0.824
20	2.29E–1	1.31E–3	1.28E–2	6.06E–4	5.56E–1	1.67E–3	7.14E–2	3.17E–3	–1.476	0.725
23	1.52E–1	1.00E–3	1.14E–2	6.95E–4	4.76E–1	1.54E–3	5.03E–2	3.86E–3	–1.885	0.598
26	1.08E–1	6.55E–4	1.09E–2	6.02E–4	4.22E–1	1.15E–3	4.61E–2	3.55E–3	–2.222	0.571
29	8.05E–2	4.49E–4	1.02E–2	5.31E–4	3.78E–1	9.00E–4	3.70E–2	3.32E–3	–2.520	0.508
32	5.94E–2	3.71E–4	9.21E–3	5.62E–4	3.42E–1	8.55E–4	3.85E–2	3.71E–3	–2.824	0.518
35	4.28E–2	3.30E–4	9.05E–3	6.76E–4	3.12E–1	9.17E–4	2.52E–2	4.66E–3	–3.152	0.415
38	2.98E–2	1.72E–4	8.42E–3	4.88E–4	2.88E–1	5.91E–4	2.76E–2	3.45E–3	–3.512	0.435
41	1.90E–2	1.63E–4	8.01E–3	7.16E–4	2.66E–1	7.56E–4	3.14E–2	5.13E–3	–3.963	0.465
44	1.15E–2	8.31E–5	7.47E–3	6.00E–4	2.49E–1	5.42E–4	4.19E–2	4.25E–3	–4.466	0.542
47	6.41E–3	7.13E–5	7.40E–3	9.55E–4	2.33E–1	7.18E–4	4.25E–2	6.55E–3	–5.051	0.546
50	3.33E–3	2.72E–5	6.36E–3	7.32E–4	2.22E–1	6.08E–4	5.66E–2	4.94E–3	–5.704	0.638
53	1.59E–3	1.55E–5	6.77E–3	9.76E–4	2.06E–1	5.69E–4	5.02E–2	5.92E–3	–6.444	0.597
56	7.39E–4	9.98E–6	7.39E–3	1.56E–3	1.96E–1	5.85E–4	4.64E–2	7.91E–3	–7.210	0.573
59	3.31E–4	8.92E–6	8.23E–3	3.39E–3	1.85E–1	6.87E–4	4.43E–2	1.31E–2	–8.014	0.559
62	1.46E–4	1.02E–5	6.58E–3	8.59E–3	1.78E–1	6.92E–4	4.98E–2	2.07E–2	–8.829	0.595

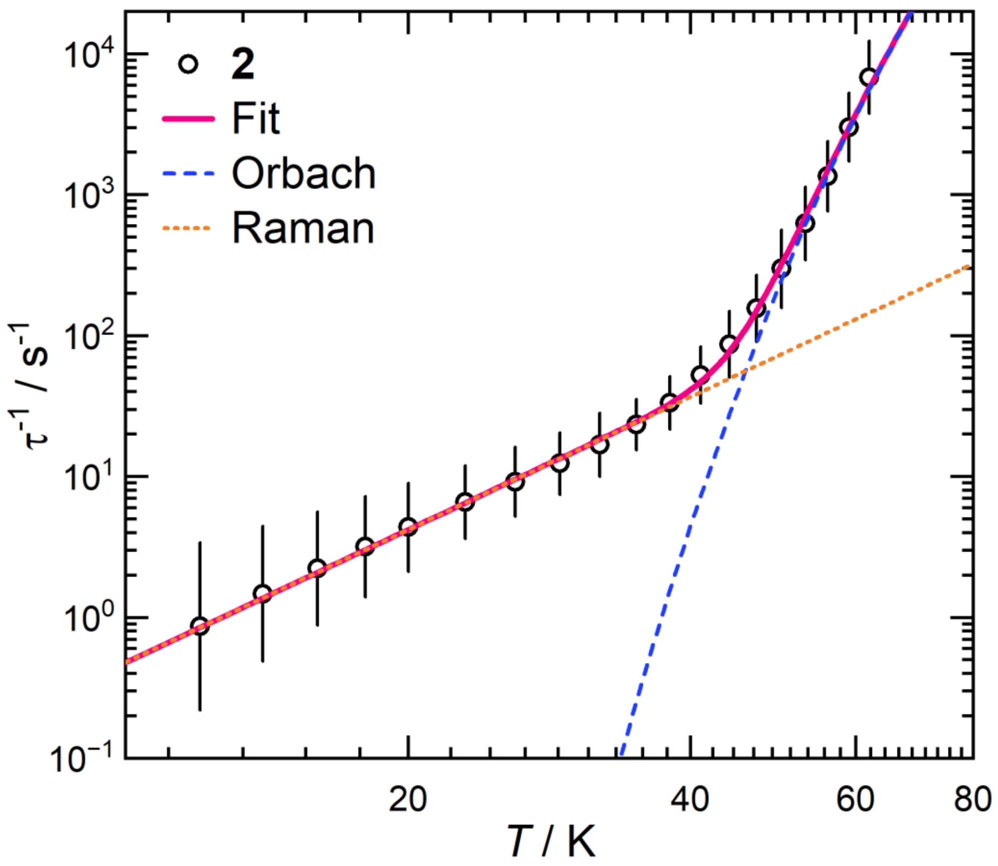

Figure S23. Relaxation profile for **2** showing contributions from Orbach and Raman processes.

5. CASSCF-SO Calculations

Table S4. Electronic structure of **1** calculated with the crystal field parameters obtained from CASSCF-SO using the solid-state geometry of **1** in zero-field. Each row corresponds to a Kramers doublet.

Energy (cm^{-1})	Energy (K)	g_x	g_y	g_z	Angle[a] (deg)	Wavefunction	$\langle J_z \rangle$
0.00	0.00	0.0002	0.0002	19.92	--	98% $\lvert\pm 15/2\rangle$	±7.469
357.76	514.81	0.005	0.005	17.0	2.2	98% $\lvert\pm 13/2\rangle$	±6.452
608.55	875.69	0.04	0.04	14.3	4.4	94% $\lvert\pm 11/2\rangle$ + 2% $\lvert\pm 7/2\rangle$	±5.454
778.57	1120.34	0.2	0.3	11.5	3.3	88% $\lvert\pm 9/2\rangle$ +6% $\lvert\pm 5/2\rangle$ + 2% $\lvert\pm 13/2\rangle$ + 2% $\lvert\pm 7/2\rangle$	±4.375
892.47	1284.25	3.8	5.3	7.4	14.1	68% $\lvert\pm 7/2\rangle$ + 18% $\lvert\pm 3/2\rangle$ + 6% $\lvert\mp 1/2\rangle$ + 3% $\lvert\pm 11/2\rangle$	±2.800
970.47	1396.49	2.4	5.0	10.9	84.0	45% $\lvert\pm 5/2\rangle$ + 26% $\lvert\pm 1/2\rangle$ + 13% $\lvert\mp 7/2\rangle$ + 6% $\lvert\mp 3/2\rangle$ + 5% $\lvert\pm 9/2\rangle$ + 3% $\lvert\pm 7/2\rangle$	±1.007
1105.25	1590.43	0.09	0.2	16.0	88.3	28% $\lvert\pm 3/2\rangle$ + 20% $\lvert\mp 5/2\rangle$ + 16% $\lvert\pm 5/2\rangle$ + 15% $\lvert\mp 3/2\rangle$ + 6% $\lvert\pm 7/2\rangle$ + 5% $\lvert\pm 1/2\rangle$ + 4% $\lvert\mp 1/2\rangle$ + 4% $\lvert\mp 7/2\rangle$	±0.157
1321.33	1901.36	0.008	0.02	19.4	88.8	29% $\lvert\pm 1/2\rangle$ + 28% $\lvert\mp 1/2\rangle$ + 18% $\lvert\pm 3/2\rangle$ + 14% $\lvert\mp 3/2\rangle$ + 6% $\lvert\pm 5/2\rangle$ + 4% $\lvert\mp 5/2\rangle$	±0.143

[a] The angle between the g_z value of the excited Kramers doublet and the ground Kramers doublet.

Table S5. Electronic structure of **2** calculated with the crystal field parameters obtained from CASSCF-SO using the solid-state geometry of **2** in zero-field. Each row corresponds to a Kramers doublet.

Energy (cm^{-1})	Energy (K)	g_x	g_y	g_z	Angle[a] (deg)	Wavefunction	$\langle J_z \rangle$
0.00	0.00	0.0001	0.0002	19.86	--	97% $\|\pm 15/2\rangle$ + 3% $\|\pm 11/2\rangle$	±7.446
340.89	490.53	0.004	0.005	16.9	2.8	95% $\|\pm 13/2\rangle$ + 4% $\|\pm 9/2\rangle$	±6.404
584.20	840.65	0.2	0.2	14.2	5.9	90% $\|\pm 11/2\rangle$ + 5% $\|\pm 7/2\rangle$ + 3% $\|\pm 9/2\rangle$ + 2% $\|\pm 15/2\rangle$	±5.365
736.51	1059.82	2.4	3.4	10.2	4.4	70% $\|\pm 9/2\rangle$ + 13% $\|\pm 5/2\rangle$ + 4% $\|\pm 1/2\rangle$ + 4% $\|\pm 13/2\rangle$ + 4% $\|\pm 7/2\rangle$ + 2% $\|\pm 3/2\rangle$	±3.916
820.75	1181.04	3.9	7.0	8.1	80.2	39% $\|\pm 7/2\rangle$ + 25% $\|\pm 3/2\rangle$ + 14% $\|\mp 1/2\rangle$ + 8% $\|\mp 9/2\rangle$ + 6% $\|\pm 11/2\rangle$ + 3% $\|\mp 5/2\rangle$ + 2% $\|\pm 5/2\rangle$ + 2% $\|\pm 9/2\rangle$	±1.664
917.08	1319.65	0.03	0.3	13.5	82.8	19% $\|\pm 7/2\rangle$ + 18% $\|\pm 5/2\rangle$ + 15% $\|\mp 7/2\rangle$ + 13% $\|\pm 1/2\rangle$ + 13% $\|\mp 5/2\rangle$ + 8% $\|\pm 9/2\rangle$ + 7% $\|\mp 1/2\rangle$ + 3% $\|\mp 9/2\rangle$	±0.584
1073.12	1544.19	0.1	0.2	16.5	88.0	24% $\|\pm 5/2\rangle$ + 19% $\|\mp 3/2\rangle$ + 18% $\|\pm 3/2\rangle$ + 15% $\|\mp 5/2\rangle$ + 8$\|\mp 7/2\rangle$ + 6% $\|\pm 7/2\rangle$ + 5% $\|\pm 1/2\rangle$ + 2 $\|\mp 1/2\rangle$	±0.189
1317.98	1896.55	0.005	0.006	19.5	88.5	28% $\|\pm 1/2\rangle$ + 26% $\|\mp 1/2\rangle$ + 19% $\|\pm 3/2\rangle$ + 14% $\|\mp 3/2\rangle$ + 7% $\|\pm 5/2\rangle$ + 4% $\|\mp 5/2\rangle$	±0.178

[a] The angle between the g_z value of the excited Kramers doublet and the ground Kramers doublet.
